# Characteristics of the smallest brucellaphage with strong lytic ability

**DOI:** 10.3389/fvets.2025.1530123

**Published:** 2025-02-05

**Authors:** Hongbaiyu Liu, Youhong Zhong, Zhihong Zhang, Kehong Xu, Chunpeng Mao, Qiuju Yang, Lihua Yang, Binbin Yu, Ying Long, Xinyu Qin, Liyuan Shi, Sheng Chang, Yuanying Shen, Peng Wang

**Affiliations:** ^1^Department of Medical Microbiology and Immunology, School of Basic Medical Sciences, Dali University, Dali, China; ^2^Yunnan Key Laboratory for Zoonosis Control and Prevention, Yunnan Institute for Endemic Disease Control and Prevention, Dali, China; ^3^Chuxiong Center for Disease Control and Prevention, Chuxiong, China; ^4^Yunnan Provincial Key Laboratory of Entomological Biopharmaceutical, College of Pharmacy, Dali University, Dali, China; ^5^School of Public Health, Kunming Medical University, Kunming, China; ^6^School of Public Health, Dali University, Dali, China

**Keywords:** phage, *Brucella*, biological characteristics, genome analysis, smallest brucellaphage

## Abstract

Brucellosis is a globally prevalent zoonotic disease caused by *Brucella* spp. posing significant threats to animal and human health. In this study, a novel lytic brucellaphage designated Y17 was isolated from sheep fecal samples collected in Ludian County, Yunnan Province, China. Transmission electron microscopy revealed that Y17 was composed of an icosahedral head (48.1 ± 2 nm) and a short tail (10.8 ± 1 nm), making it the smallest brucellaphage described so far. The optimal multiplicity of infection (MOI) for phage Y17 is 0.001, with a burst size of ~187 PFU/cell, the largest value reported for any brucellaphage, and it has a relatively short latent period. It exhibits broad pH and temperature stability, retaining activity even after 1 h of exposure to ultraviolet radiation and various ethanol concentrations. Y17 shows strong lytic activity against *Brucella abortus* and can also infect some *Brucella melitensis* strains. The Y17 genome spans 38,025 bp with a GC content of 48.2%, making it the smallest genome among brucellaphages to date. It lacks virulence, antibiotic resistance, or lysogenic genes, indicating its potential as a safe biocontrol agent. Whole-genome average nucleotide identity (ANI) analysis reveals high homology across all lytic brucellaphages, but Y17 exhibits relatively lower genome coverage compared to other lytic brucellaphages. Genomic collinearity comparison revealed that Y17 lacks some terminal fragments present in the genomes of other lytic brucellaphages. Furthermore, compared to brucellaphages with genomes larger than 40 kb, Y17 also lacks segments corresponding to ORF21 (amidase), ORF28 (hypothetical protein), and ORF29 (carbohydrate-binding protein). Phylogenetic analysis indicates that Y17 is closely related to phages Iz, Bk2, S708, Wb, R/C, Pr, and Bk. Moreover, the capsid gene shows significantly higher conservation in comparison with the tail collar and amidase genes. This study significantly enriches the brucellaphage database and highlights the potential of Y17 as a biocontrol agent for managing brucellosis in endemic regions.

## 1 Introduction

*Brucella* are highly infectious, facultatively intracellular bacterial pathogens that cause reproductive failure and abortion in infected animals. Humans are typically infected through direct contact with infected animals, ingestion of contaminated animal products, or inhalation of airborne pathogens. Therefore, brucellosis causes significant economic losses in livestock and poses a serious threat to human health, particularly in developing countries where livestock management practices are insufficiently regulated (1). Currently, *Brucella* is classified into 12 main species (http://www.bacterio.net/brucella.html), and different species exhibit close genetic relationships, with ~97% similarity at the genome level. However, distinct *Brucella* species present different host preferences, zoonotic risks, and levels of virulence (2). The most common species of *Brucella* in livestock are *Brucella melitensis, Brucella suis, Brucella abortus* and *Brucella canis*, with *Brucella melitensis* being the most contagious and pathogenic among them (3). Brucellosis primarily affects the host reproductive system. In the acute phase, infection often presents with fever, which can persist and progress into a chronic, disabling disease with systemic complications (4). The treatment for brucellosis typically involves a combination of multiple antibiotics; however, complete eradication is challenging, and the disease often becomes chronic. In recent years, the emergence of an increasing number of antibiotic-resistant strains has posed even greater challenges to brucellosis treatment (5).

Bacteriophages (phages) are viruses that exclusively infect bacteria and are the most abundant biological entities on earth. They are commonly found in seawater, soil, and the gastrointestinal tracts of animals and play crucial roles in maintaining the microbial ecological balance of the planet (6). With the advent of the postantibiotic era, bacteriophages are increasingly regarded as having great potential for the treatment of bacterial infections (7). *Brucella* bacteriophages were first isolated in the 1950s. Most of the brucellaphages described to date are lytic and host specific, primarily infecting *B. abortus* (8). The phage BiPBO1 is a member of the family Siphoviridae and is the only reported temperate brucellaphage (9). Lytic brucellaphages are very similar to each other in terms of morphology, antigenic reactions, and overall physicochemical properties. These phages exhibit podoviral morphology and have double-stranded DNA as their genetic material. Due to the strong DNA homology among their genomes, they are considered a single taxonomic species (8, 10). However, lytic brucellaphages still exhibit minor differences in particle size, host range, and other biological characteristics (11). Since the isolation of phage Tb in Tbilisi, Georgia, in 1955, phages have been commonly used for the identification and diagnosis of *Brucella* species (12). To date, brucellaphages have been classified into six groups on the basis of their host range, represented by the diagnostic phages: Tb, Wb, Bk, Fi, Iz, and R/C (13). Moreover, three major genetic groups were subsequently identified based on the genomic homology of six diagnostic brucellaphages, including Tb, Fz, Wb, S708, Bk, and R/C, and these groups were consistent with their defined host range phenotypes. Group I includes the Tb and Fz phages, which are mainly lytic for *B. abortus* and *B. neotomae*;Group II comprises the Bk, R/C, and Pr phages, which are predominantly lytic for *B. abortus, B. melitensis* and *B. suis*;and Group III is constituted by phages Wb and S708, which are lytic for *B. suis, B. abortus*, and *B. neotomae* (14). Because bacteriophages typically exhibit specific lytic activity against their host bacteria, the isolation of brucellaphages is important not only for the epidemiological assessment of brucellosis but also for their potential application in the treatment and prevention of this disease.

In this study, we successfully isolated a novel lytic brucellaphage, named Y17, from sheep feces collected in Ludian County, Yunnan Province, China. Phage Y17 is the smallest brucellaphage reported to date, in terms of both particle and genome size. When infecting *Brucella*, it demonstrates a significantly higher burst size compared to other reported brucellaphages, highlighting its potential for applications in biotechnology, such as phage therapy or environmental disinfection. Here, we provide a comprehensive analysis of the biological properties and genomic features of Y17, emphasizing its unique characteristics and potential value in combating brucellosis.

## 2 Materials and methods

### 2.1 Growth media for bacteria and phages

The *Brucella abortus* A19 vaccine strain was used as the indicator strain for phage isolation. This strain was obtained from the Yunnan Institute of Endemic Disease Control and Prevention (YIEDC). Thirty clinical isolates of *Brucella* were collected from Kunming, Yuxi, and Qujing cities in Yunnan Province, all bacterial strains were preserved at the YIEDC. The *Brucella abortus* 104M vaccine strain, the *Brucella melitensis* M5 vaccine strain, and the diagnostic phage Tb were obtained from the Chinese Center for Disease Control and Prevention (CCDC). For all experiments, *Brucella* was cultured on *Brucella* agar (BD, USA) and propagated in *Brucella* broth (BD, USA). The strains were stored at −80°C in 25% glycerol and grown at 37°C under standard aerobic incubation conditions.

### 2.2 Phage isolation and purification

The collected sheep fecal samples were soaked in SM buffer (5.8 g/L NaCl, 2.0 g/L MgSO_4_, 50 ml/L of 1 M Tris, pH 7.5, and 5 ml/L of presterilized 2% gelatin) at room temperature for 24 h. For phage enrichment, 5 ml of the sample supernatant was mixed with 500 μl of the A19 vaccine strain and 10 ml of *Brucella* broth, followed by continuous shaking at 220 rpm at 37°C for 48 h. The mixture was then centrifuged at 6,000*g* for 10 min and filtered through a 0.22 μm filter to obtain the phage suspension. The A19 strain was mixed with the phage suspension and plated on a double-layer agar plate. After incubation at 37°C for 24–48 h, individual plaques were picked from the plate and propagated. The double-layer agar plate method was repeated three times to obtain a purified phage suspension.

### 2.3 Transmission electron microscopy

The purified phages were placed onto 400 mesh carbon-coated grids and negatively stained with 2% phosphotungstic acid (pH 6.5). The morphology of the phages was then observed using a Hitachi HT7700 transmission electron microscope. The dimensions of three viral particles of each phage were measured, and the measurements were averaged.

### 2.4 Multiplicity of infection (MOI) experiment

The phage and host bacterial suspensions were mixed at specific MOIs (0.0001, 0.001, 0.01, 0.1, 1, and 10) and incubated at 37°C with shaking at 220 rpm for 24 h. The mixture was then passed through a 0.22 μm filter to remove bacteria. The phage titer was determined using the double-layer agar method, and the MOI that produced the highest phage titer was considered the optimal MOI.

### 2.5 One-step growth curve

The one-step growth curve of phage Y17 was generated according to a previously described method with minor modifications (15). The host bacteria and phage were mixed to achieve an MOI of 0.001, and the mixture was incubated at 37°C for 15 min. Afterward, the mixture was centrifuged at 10,000*g* for 1 min, and the pellet was resuspended in 1 ml of *Brucella* broth. The supernatant was discarded, and the pellet was washed 2–3 times to remove any unadsorbed phages. The pellet was then resuspended in 8 ml of *Brucella* broth and incubated at 37°C with shaking at 220 rpm for 2 h. At 10-min intervals, 500 μL samples were collected and immediately centrifuged at 10,000*g* for 1 min to pellet the cells, after which the phage titer in the supernatant was determined. The burst size was calculated as the ratio of the final phage titer to the number of initial bacterial cells infected (16).

### 2.6 Stability studies

The stability of the phage was tested under various conditions by treating a 10^10^ pfu/ml phage suspension for 1 h at different pH levels (0–14), UV irradiation (15 cm from the UV lamp, samples were taken every 5 min during exposure), temperatures (4, 28, 37, 42, 50, 60, 70, and 80°C), and ethanol concentrations (10%, 25%, 50%, 75%, and 95%). After treatment, the phage titer was determined via the double-layer agar method, followed by incubation at 37°C for 24–48 h.

### 2.7 Host range analysis

In this study, the A19, 104M, and M5 vaccine strains, along with 30 clinical isolates of *B. melitensis* (as detailed in Table 1), were selected as the experimental strains. The spot test method was used to determine the host range of phages Y17 and Tb, and the lytic effect was evaluated after incubation at 28 or 37°C for 48 h.

**Table 1:** The host range of phage Y17 and Tb.

**Number**	**Source**	**Strain name**	**Species**	**Biovar**	**Y17**		**Tb**	
					**28**°**C**	**37**°**C**	**28**°**C**	**37**°**C**
1	Qujing	202001	*Brucella melitensis*	3	+	±	–	–
2	Yuxi	202038	*Brucella melitensis*	3	±	–	–	–
3	Kunming	202035	*Brucella melitensis*	N	–	–	–	–
4	Kunming	202008	*Brucella melitensis*	N	–	–	–	–
5	Kunming	202043	*Brucella melitensis*	3	+	±	–	–
6	Yuxi	202022	*Brucella melitensis*	3	–	–	–	–
7	Qujing	202019	*Brucella melitensis*	3	+	±	±	–
8	Qujing	202037	*Brucella melitensis*	1	±	±	–	±
9	Kunming	202012	*Brucella melitensis*	N	–	–	–	–
10	Kunming	202047	*Brucella melitensis*	N	–	–	–	–
11	Kunming	202093	*Brucella melitensis*	N	–	–	–	–
12	Kunming	202007	*Brucella melitensis*	1	±	±	–	–
13	Kunming	202045	*Brucella melitensis*	3	±	–	–	–
14	Qujing	202122	*Brucella melitensis*	N	±	±	–	–
15	Qujing	202176	*Brucella melitensis*	3	+	+	–	–
16	Yuxi	202166	*Brucella melitensis*	3	±	±	–	–
17	Kunming	202138	*Brucella melitensis*	N	±	±	–	±
18	Kunming	202185	*Brucella melitensis*	3	±	–	–	–
19	Kunming	202120	*Brucella melitensis*	N	–	–	–	–
20	Qujing	202143	*Brucella melitensis*	3	–	–	–	–
21	Kunming	2021103	*Brucella melitensis*	N	±	–	–	–
22	Yuxi	202197	*Brucella melitensis*	3	±	–	–	–
23	Qujing	2021101	*Brucella melitensis*	N	±	–	–	–
24	Kunming	202170	*Brucella melitensis*	3	–	–	–	–
25	Kunming	202139	*Brucella melitensis*	N	–	–	–	–
26	Qujing	202144	*Brucella melitensis*	3	–	–	–	–
27	Qujing	2021105	*Brucella melitensis*	N	+	±	–	±
28	Yuxi	202188	*Brucella melitensis*	3	–	–	–	–
29	Kunming	202198	*Brucella melitensis*	3	±	±	–	–
30	Kunming	202157	*Brucella melitensis*	3	±	±	–	–
31	CCDC	M5	*Brucella melitensis*	–	±	±	–	–
32	CCDC	104M	*Brucella abortus*	–	+	+	+	+
33	YIEDC	A19	*Brucella abortus*	–	+	+	+	+

+, all lysis, plaque is clear; ±, semi-lytic, plaque is unclear; –, no lysis; N, nottested. Strains numbered 1–30 are Brucella strains isolated from clinical samples, while strains numbered 31–33 are Brucella vaccine strains.

### 2.8 Genome sequencing and bioinformatics analysis

Phage genomic DNA was extracted using the Phage Genomic DNA Extraction Kit (Abigen Corp, Beijing, China). The DNA was fragmented to the desired length, and an “A” base was added to the 3′ ends to enable ligation with adapters containing a complementary “T” base. PCR amplification of adapter-ligated DNA fragments was performed to construct the library, which was then sequenced using the Illumina NovaSeq X Plus platform. Quality control was conducted using Soapnuke (v2.0.5) to generate high-quality clean reads (17). BWA (v0.7.17) aligned reads to the host genome and filtered out host-derived sequences (18). *De novo* assembly was conducted with Megahit (v1.1.2 SCIME) (19). Circular genomes were identified using ccfind (v1.4.5), with overlapping terminal sequences trimmed.

The putative genes in the phage genome were identified using the RAST server (20). The whole genome sequence of the phage was aligned using NCBI BLAST+ software (v 2.16.0) to further predict the function of each putative gene. The genome map was generated with SnapGene (v6.0.2). PhageLeads was used to check the genome for temperate markers, antibiotic resistance genes, and virulence genes (21). FastANI software was used to conduct pairwise comparisons of phage genomes at the nucleotide level by calculating their average nucleotide identity (ANI) (22). Comparative genomic maps were constructed using EasyFig (v2.2.5) (23). A whole-genome phylogenetic tree was constructed with OrthoFinder (24), and a protein phylogenetic tree was constructed using MEGA 11. Both trees were constructed using the maximum likelihood method. The initial multiple sequence alignment of amino acids was generated using MEGA, and the results were subsequently visualized and analyzed with ESPript 3.0 (25).

## 3 Results

### 3.1 The biological characteristics of phage Y17

**Figure 1:**
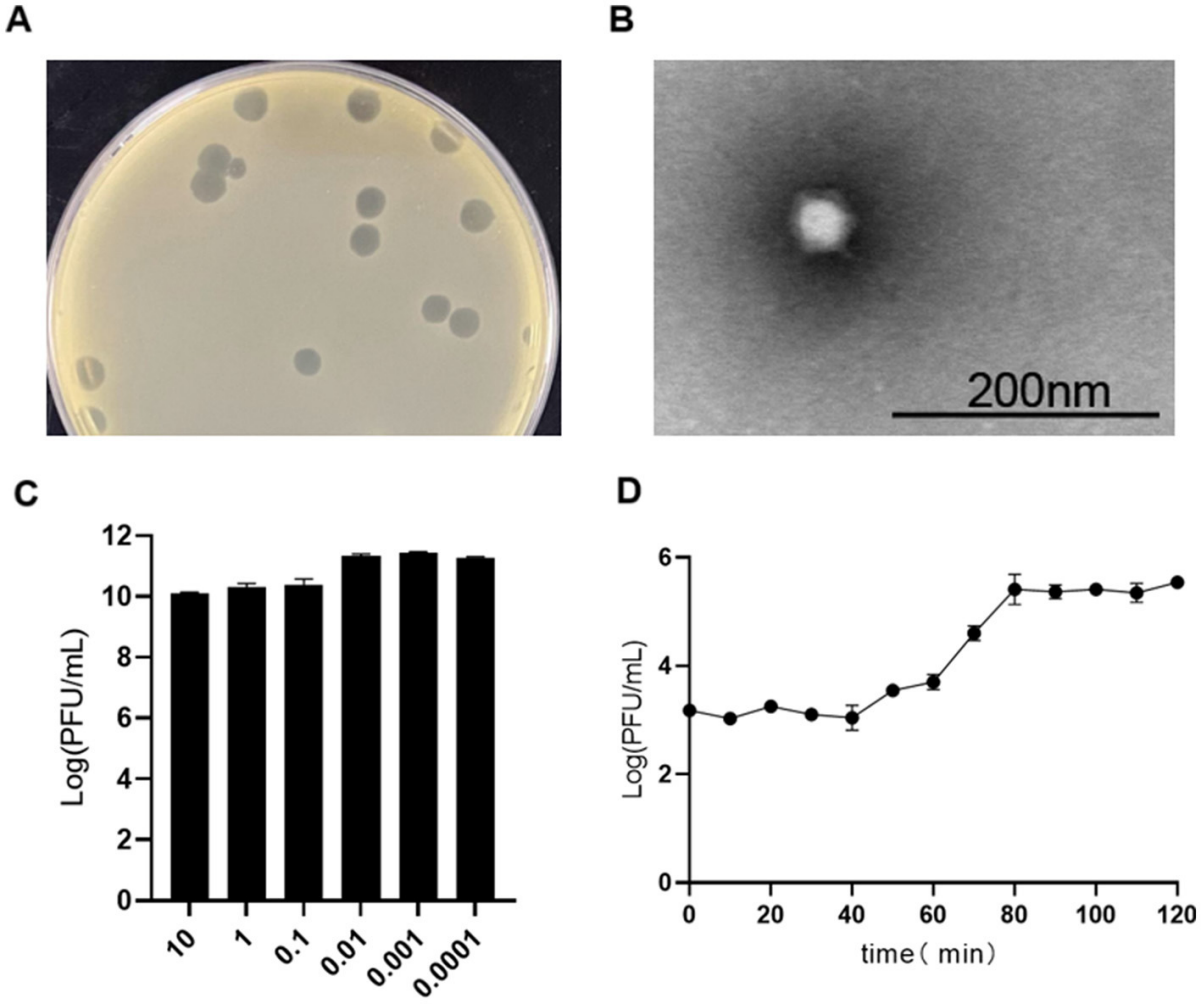
Biological characteristics of phage Y17. **(A)** Plaque morphology, **(B)** transmission electron micrograph, **(C)** optimal multiplicity of infection (MOI), and **(D)** one-step growth curve. Error bars represent the standard deviation of the mean.

Brucellaphage Y17, isolated from sheep fecal samples in Yunnan Province, forms clear plaques measuring 3–6 mm in diameter on the A19 vaccine strain in double-layer agar (Figure 1A). Transmission electron microscopy (TEM) revealed that phage Y17 was composed of an icosahedral head measuring 48.1 ± 2 nm and a short, noncontracted tail measuring 10.8 ± 1 nm (Figure 1B). Phage Y17 exhibited the highest infection efficiency at an MOI of 0.001 (Figure 1C). The one-step growth curve showed that the efficacy of phage Y17 significantly increased after 40 min and stabilized at 80 min, with an estimated burst size of ~187 pfu/cell (Figure 1D).

We tested the host ranges of the phages Y17 and Tb in 30 clinical isolates of *B. melitensis* and the M5 and 104M vaccine strains. Both Y17 and Tb demonstrated significant lytic activity against *B. abortus*. However, Tb exhibited little lytic activity against *B. melitensis*. In contrast, Y17 showed some lytic activity against most *B. melitensis* strains, but clear plaques were observed in only a few strains. Additionally, the lytic effect of Y17 was greater at 28°C than at 37°C. These findings indicate that these brucellaphages exhibit host specificity (Table 1).

### 3.2 Stability studies

**Figure 2:**
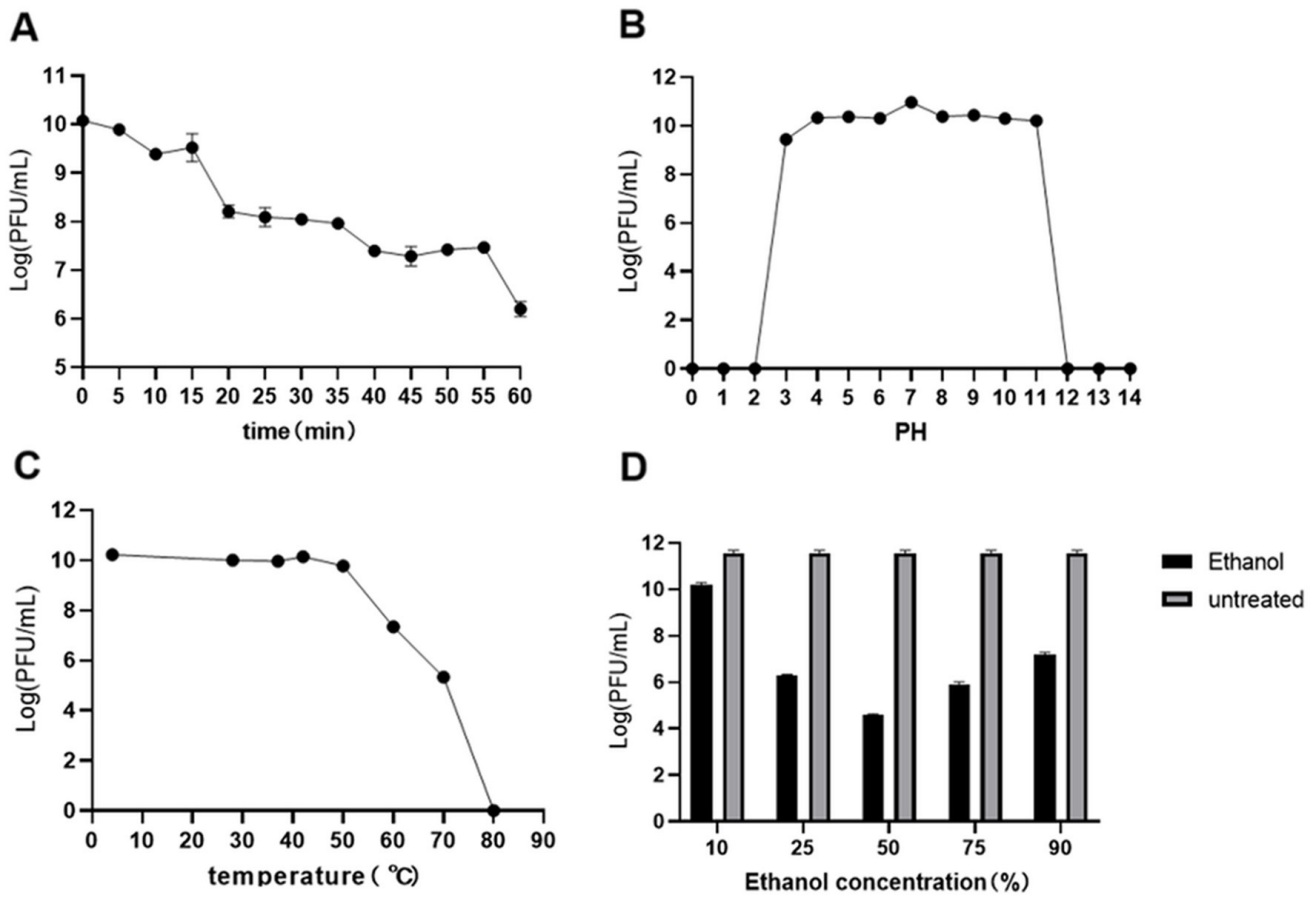
**(A)** Utraviolet radiation, **(B)** pH, **(C)** temperature, and **(D)** ethanol stability of phage Y17. Error bars represent the standard deviation of the mean.

After 1 h of UV irradiation, the titer of phage Y17 gradually decreased but retained significant activity (Figure 2A). The optimal pH for Y17 was 7. Its titer remained high and relatively stable within the pH range of 3 to 11. Phage activity was completely lost only at pH ≤ 2 or ≥12, indicating a broad pH tolerance (Figure 2B). Phage Y17 retained stable activity below 50°C, but was completely inactivated at 80°C (Figure 2C). Exposure of Y17 to various ethanol concentrations for 1 h did not cause complete inactivation, suggesting that Y17 is a non-enveloped phage (Figure 2D).

### 3.3 Whole-genome analysis of phage Y17

**Figure 3:**
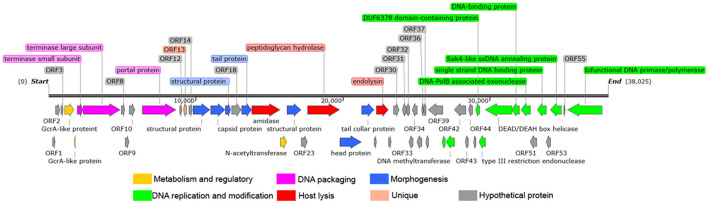
Whole genome map of Y17. The direction of the arrow represents transcription orientation. Colored boxes represent the functional classification or unique features of the corresponding genes.

The Y17 genome comprises a 38,025 bp circular double-stranded DNA, and no tRNA genes were predicted. The GC content is 48.2%, lower than the 57% observed in its host *Brucella* strains (26). An online whole-genome BLASTn search revealed that phage Y17 has over 99% coverage and identity with all other lytic brucellaphages, indicating a close relationship between Y17 and other lytic brucellaphages. The RAST Server predicted a total of 56 ORFs in the Y17 genome. Among them, 31 ORFs are located on the positive strand, primarily distributed in the beginning and middle regions. Meanwhile, 25 ORFs are on the negative strand, concentrated toward the end of the genome. Each ORF of Y17 was analyzed using NCBI BLAST+ software (v 2.16.0) against all proteins of other lytic brucellaphages recorded in NCBI. The analysis predicted 26 functional proteins, while the remaining 30 were identified as hypothetical proteins. Notably, one predicted gene product of unknown function exhibited no homology to any published sequences in the database (Figure 3; Supplementary Tables S1, S2). Additionally, PhageLeads was used to screen the genome and identified no genes associated with the temperate life cycle, antibiotic resistance, or bacterial virulence. These findings highlight the safety and therapeutic potential of Y17, supporting its application in clinical and environmental settings (21).

The 26 predicted functional proteins were categorized into five functional modules: metabolism and regulatory, DNA packaging, structure, host lysis, and DNA replication and modification. The metabolism and regulation-related proteins included GcrA-like proteins (ORF4 and ORF5) and N-acetyltransferase (ORF21). Although the specific functions of these proteins remain unknown, they may play a role in genome expression and the transcriptional regulation of the host bacterium during infection (27, 28). Several proteins involved in DNA packaging work together to ensure that the DNA is efficiently and accurately packaged into the viral capsid (29). The DNA packaging proteins in Y17 include the small terminase subunit (ORF6), the large terminase subunit (ORF7), and the portal protein (ORF11). The morphogenesis-related proteins include the capsid protein (ORF16), structural proteins (ORF15, ORF17, and ORF22), tail protein (ORF19), head protein (ORF25), and tail collar protein (ORF26). The tail collar protein is likely involved in the host specificity of the phage (30, 31). Amidase (ORF20), peptidoglycan hydrolase (ORF24), and endolysin (ORF28) are likely involved in host lysis. Holin was not identified in the lysis module. The DNA replication and modification module is located at the end of the genome and include the following: DNA methyltransferase (ORF40 and ORF41), DUF6378 domain-containing protein (ORF46), type III restriction endonuclease (ORF47), DEAD/DEAH box helicase (ORF48), DNA-binding protein (ORF49), DNA-PolB associated exonuclease (ORF50), single strand DNA binding protein (ORF52), Sak4-like ssDNA annealing protein (ORF54), and bifunctional DNA primase/polymerase (ORF56).

### 3.4 Homology and average nucleotide identity (ANI)

**Figure 4:**
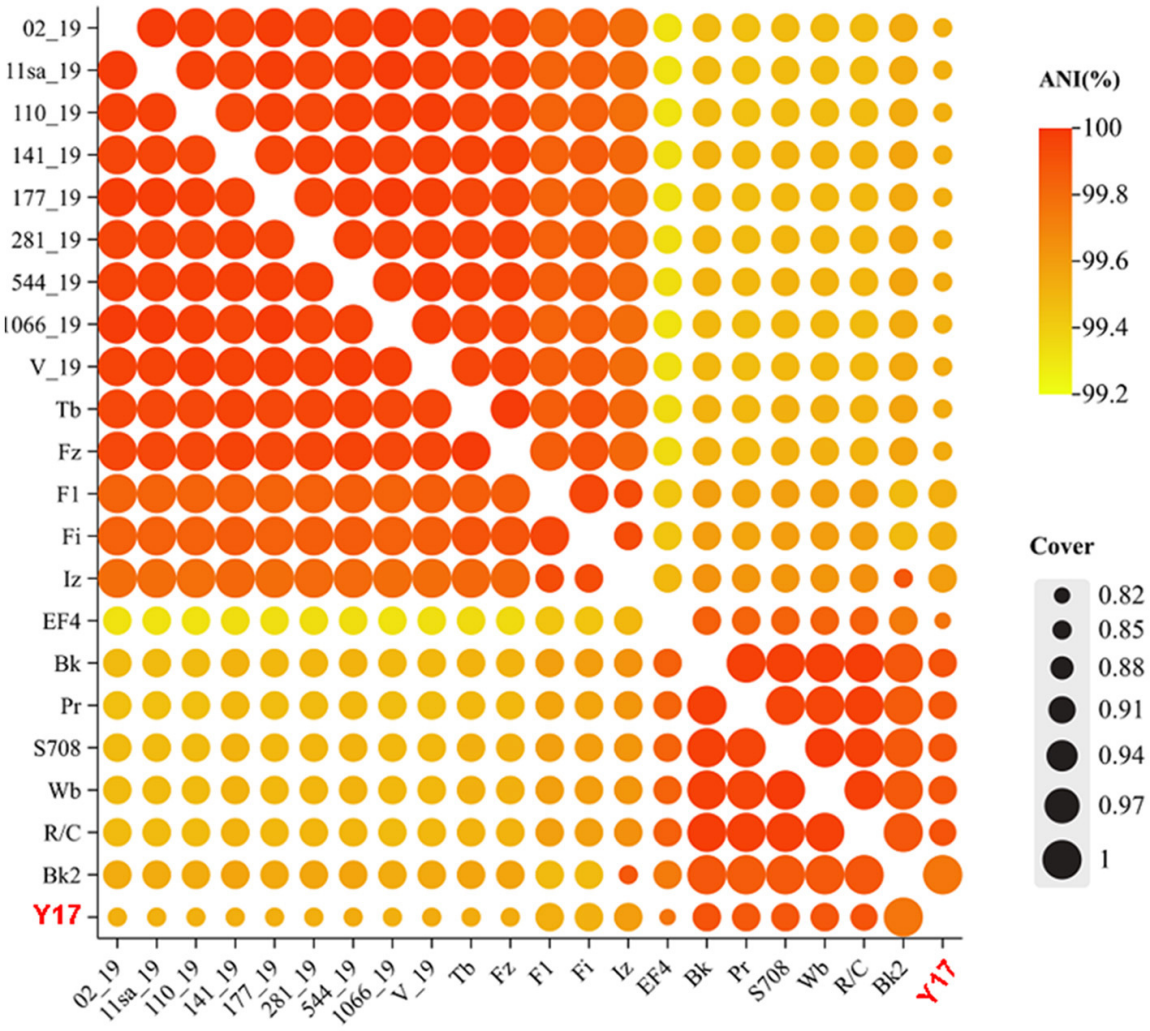
Homology and average nucleotide identity (ANI).

The ANI heatmap of Y17 and all lytic brucellaphages in NCBI (a total of 21 phages) is shown in Figure 4. There is a high level of genomic similarity among all brucellaphages, with ANI values ranging from 99.32% to 100%, as calculated using FastANI (Supplementary Table S3). This indicates a high degree of genomic conservation among these phages. Y17 exhibited the lowest overall nucleotide coverage, with the majority of coverage values ranging from 0.85 to 0.92, suggesting potential divergence in genome. The highest coverage of 1 was exclusively observed with Bk2, likely due to a close evolutionary relationship (Supplementary Table S4).

### 3.5 Genomic collinearity comparison

**Figure 5:**
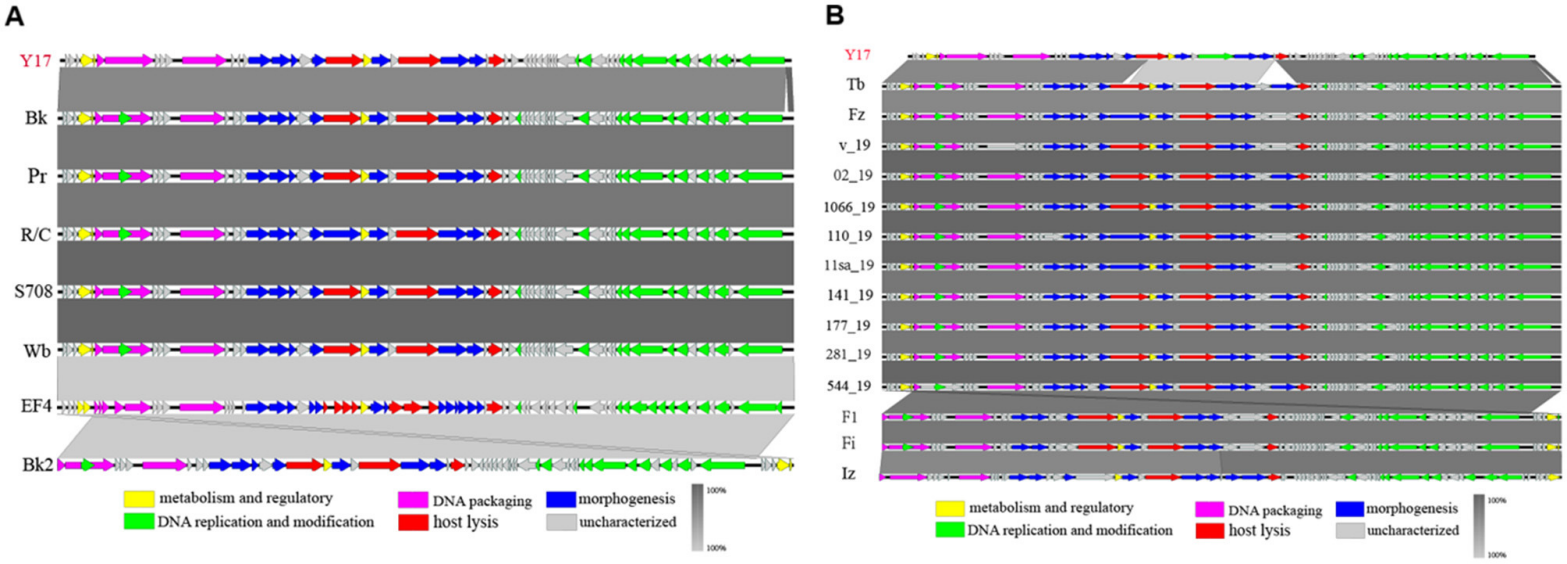
The easyfig output illustrates the genomic collinearity analysis. **(A)** between Y17 and brucellaphages with genomes smaller than 40 kb, and **(B)** those with genomes larger than 40 kb. Predicted functional proteins are represented in different colors, with arrows indicating gene length and transcription orientation. Shading beneath the genomes represents sequence similarity, with the color intensity reflecting the degree of similarity.

We selected phage Y17 and all lytic brucellaphages for multiple collinearity comparison analyses. The comparisons were divided into two groups based on phage genome similarity: genomes smaller than 40 kb (Figure 5A) and genomes larger than 40 kb (Figure 5B).

In phages with genome lengths smaller than 40 kb, collinearity analysis revealed complete genome collinearity among Bk, Pr, R/C, S708, Wb, EF4, and Bk2. Although Y17 lacked a portion of the terminal segment, the rest of its genome also displayed complete collinearity with the others. Additionally, except for the last five ORFs of Bk2, which showed reverse homology with other phage genomes, the genomic arrangement of the remaining phages was consistent (Figure 5A).

For phages with genome lengths larger than 40 kb, collinearity analysis revealed complete genome collinearity among Tb, Fz, V_19, 02_19, 1066_19, 110_19, 11sa_19, 141_19, 177_19, 281_19, 544_19, F1, Fi, and Iz. These phages contained three major insertions relative to Y17: a partial fragment of ORF21 (amidase), ORF28 (hypothetical protein), ORF29 (carbohydrate-binding protein), and a portion of the terminal segment. Meanwhile, F1, Fi, and Iz showed reverse homology in their terminal genome segments, while the genomic arrangement of other phages remained consistent (Figure 5B).

### 3.6 Phylogenetic tree analysis

**Figure 6:**
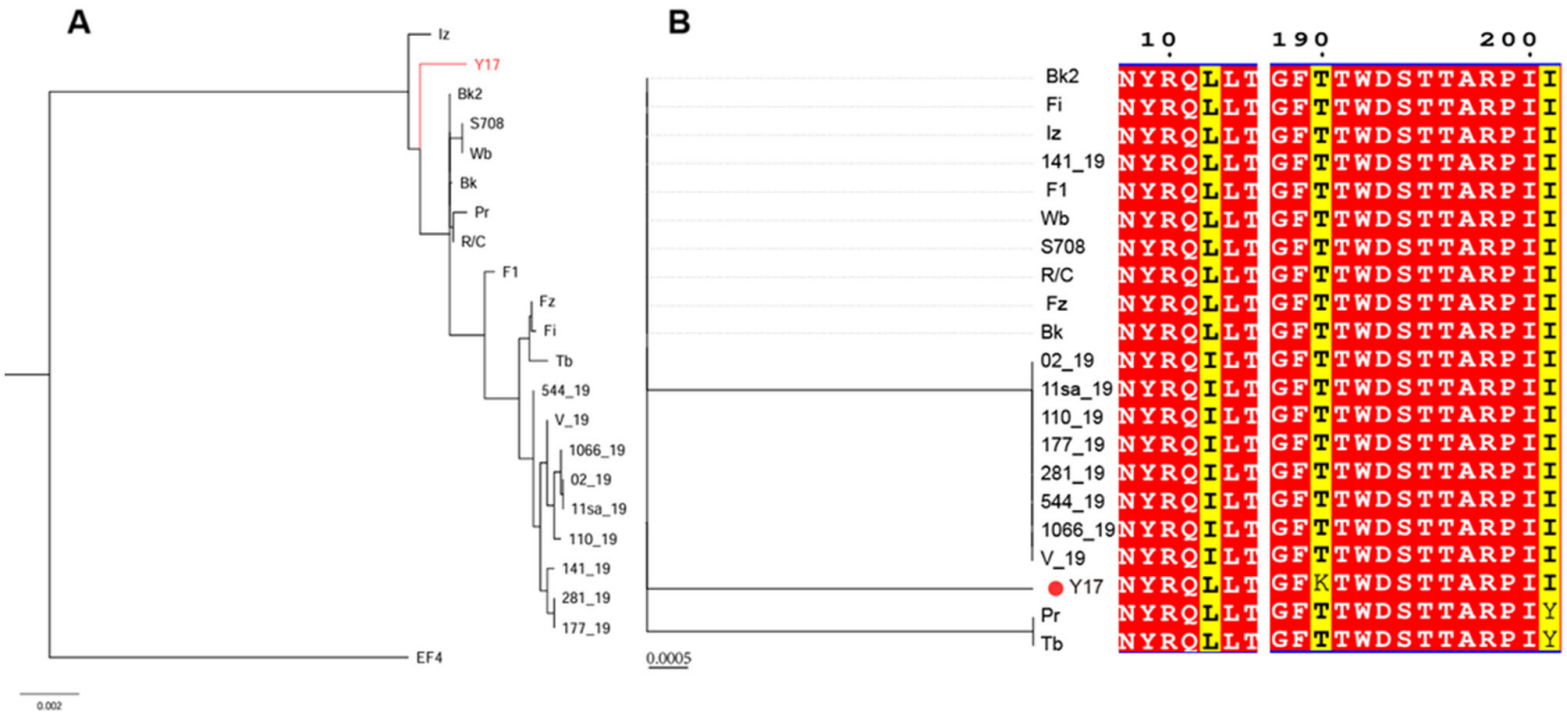
**(A)** The phylogenetic tree of Y17 and lytic brucellaphages was constructed based on whole-genome amino acid sequences. **(B)** The left panel shows the phylogenetic tree constructed based on the capsid proteins of brucellaphages, while the right panel displays the amino acid sequence alignment corresponding to each phage in the phylogenetic tree. Red indicates highly conserved amino acids, and yellow highlights key variable regions.

The phylogenetic tree based on the whole genomes of lytic brucellaphages reveals two distinct clades. EF4 forms a separate clade, indicating a more distant evolutionary relationship with other phages. Y17 clusters closely with phages Iz, Bk2, S708, Wb, R/C, Pr, and Bk, indicating a close evolutionary relationship (Figure 6A).

**Figure 7:**
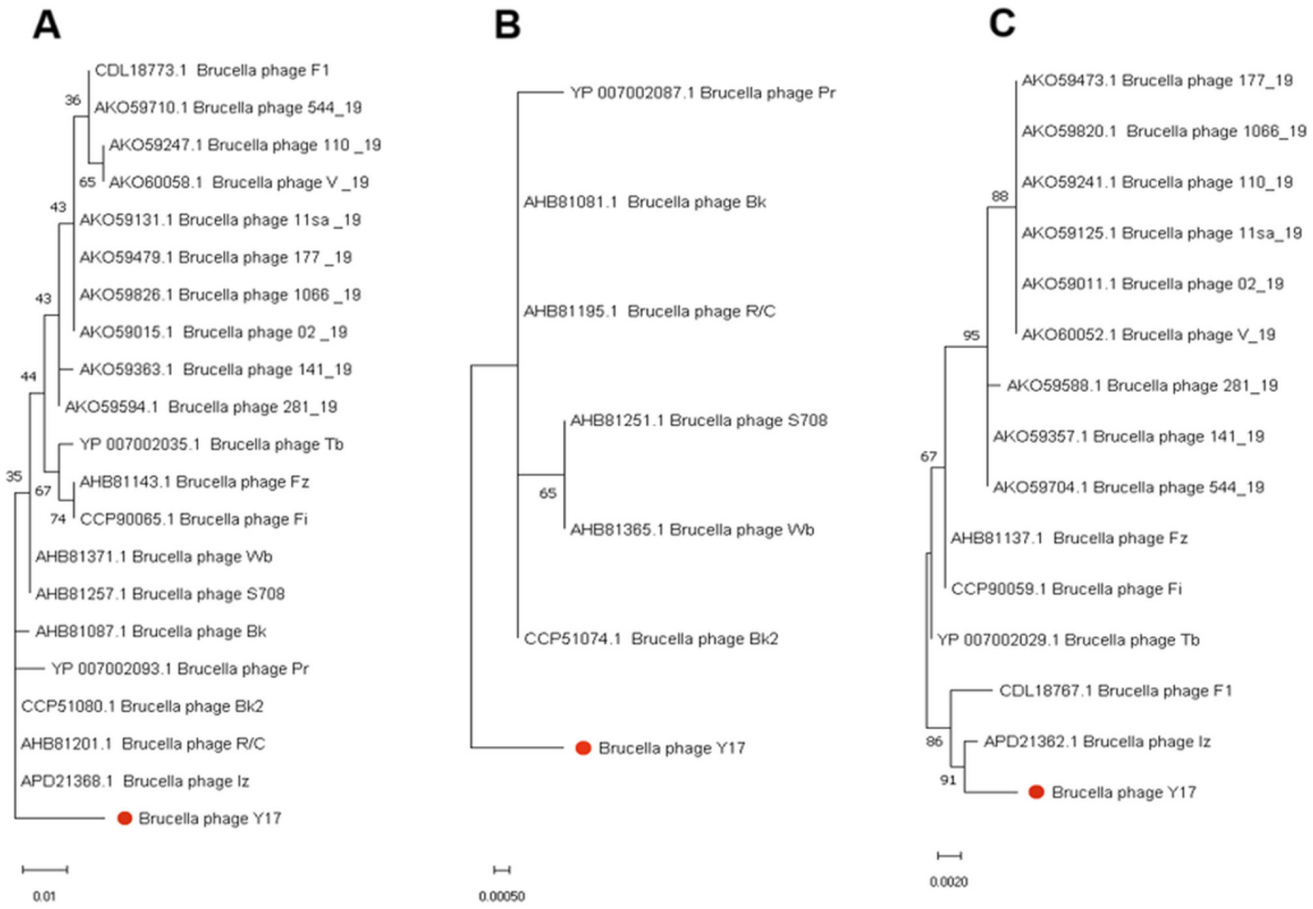
Phylogenetic trees were constructed using MEGA 11 based on the amino acid sequences of **(A)** tail collar protein, **(B)** the amidase of phages with whole genomes smaller than 40 kb, and **(C)** the amidase of phages with whole genomes larger than 40 kb.

To gain deeper insights into the evolutionary relationships of phages at different protein levels, we constructed phylogenetic trees using the amino acid sequences of the capsid protein (Figure 6B), tail collar protein (Figure 7A) and amidase to explore phage evolution. Phages with genomes smaller than 40 kb have shorter amidase sequences than those with larger genomes. Therefore, they were divided into two groups when constructing the phylogenetic tree based on amidase sequence similarity (Figures 7B, C). We excluded phage EF4 from the analysis due to its significant sequence divergence. Phage EF4 was excluded from the analysis due to its significant sequence divergence. Multiple sequence alignment of phage capsid proteins revealed high conservation, with only three variable sites. Notably, only phage Y17 exhibited amino acid variation at position 190, while all other phages remained conserved at this site (Figure 6B). The topological structures of the phylogenetic trees for the capsid protein, tail collar protein, and amidase revealed distinct variations. Among these, the evolutionary relationships of the capsid protein were the most conserved, whereas the tail collar protein displayed greater genetic diversity than the other two proteins. Notably, Y17 occupied a unique position in all three phylogenetic trees, suggesting its distinct adaptations to varying environmental pressures.

## 4 Discussion

The most notable feature of larger phages, compared to smaller ones, is their larger particle size and genome. Previously reported lytic brucellaphages have genome sizes ranging from 38.25 to 41.14 kb, with phage Iz possessing the largest genome among all lytic brucellaphages at 41,446 bp. The head diameter of brucellaphages ranges from 50 to 80 nm, while their tail lengths range from 15 to 30 nm. Notably, phage Iz has a longer tail, measuring ~40 nm (32, 33). Most brucellaphages, such as Tb, Bk, and Wb, have a head diameter of ~60 nm and a tail length of around 20 nm (11, 33, 34). In this study, brucellaphage Y17 was found to possess a genome length of 38,025 bp, a head diameter of ~48.1 nm, and a tail length of ~10.8 nm, making it the smallest brucellaphage discovered to date in terms of both genome size and particle dimensions. Smaller phages are generally more dependent on host functions during replication due to their reduced gene content. However, this genome simplification may enhance their efficiency in infecting host bacteria and replicating (35). According to the one-step growth curve assay, the latent period of phage Y17 is 40 min, with a burst size of ~187 pfu/cell. In contrast, previously reported brucellaphages have latent periods of 1 h or longer and burst sizes ranging from 22 to 121 pfu/cell (11, 30). The higher lytic efficiency of Y17 against its host bacteria is likely attributable to its smaller genome, which allows for more efficient replication during infection.

Capsid protein is an essential component of phage particle formation, and mutations in its amino acids may significantly affect the three-dimensional structure of the protein. Interestingly, in the multiple sequence alignment of capsid proteins, we observed that Y17 exhibits a mutation at the 190th amino acid position compared to other phages. This mutation may alter the local spatial conformation of the capsid protein, resulting in a more compact particle structure with a smaller head diameter. Additionally, the compact capsid structure may enhance the physical stability of the phage particle, providing better protection for the genome and enabling more efficient genome release during host infection. However, experimental evidence is required to test this hypothesis.

Phage genomes are typically packaged in either linear or circular forms, with each configuration exhibiting distinct implications for stability, replication efficiency, and infection dynamics. For example, phage Y17 contains a covalently closed circular genome without free ends, which significantly enhances its genomic stability (36). It achieves efficient replication and packaging through rolling-circle replication, which enhances genome stability and adaptability (37). In contrast, linear genomes, such as those of the T4 phage, are typically characterized by terminal redundancy or cohesive ends, making them more vulnerable to damage and degradation (38). Furthermore, linear genomes are prone to homologous recombination or crossover recombination, which promotes genetic diversity but may also lead to genome instability (39).

To date, the genomes of all reported lytic brucellaphages exhibit strong homology, with a GC content of 48%, and their genome sequence arrangements are largely consistent. Traditionally, it was believed that lytic strains isolated from different locations and at different times would not exhibit nucleotide similarity, even when infecting the same host. However, this high genomic homogeneity suggests that brucellaphages are among the most conserved phages. This unique characteristic, in contrast to the rich genomic diversity observed in other phages, may be linked to their adaptation to hosts with similar genomes (40). The genes responsible for nucleic acid replication and metabolism in brucellaphages are encoded on the negative strand, while the remaining proteins are encoded on the positive strand. This gene localization may contribute to the regulation of gene expression. Due to the high similarity of the large terminase subunits among brucellaphages, constructing a phylogenetic tree based on these subunits was not feasible. Therefore, in this study, the capsid protein, tail collar protein and amidase were selected for phylogenetic analysis. Notably, the tail collar protein and amidase exhibit greater genetic diversity and are likely to play key roles in the host selection of the phages (8). Holin, typically formed in the later stages of phage infection, disrupts the host cell membrane to facilitate the release of phage particles (41). However, no holin has been identified in brucellaphages, possibly because holins have not yet been specifically predicted in these phages or because an alternative lysis mechanism may be involved. Compared to other brucellaphages, phage Y17 lacks not only the fragments identified as missing through genomic collinearity analysis but also two essential functional proteins: the HNH endonuclease and the replication initiation factor. Phage Y17 may have evolved alternative mechanisms to compensate for these absences, such as utilizing host replication initiation factors or other auxiliary proteins to complete its replication.

While annotating the functions of each protein in Y17, we also systematically reviewed the genomes of all lytic brucellaphages and identified three major issues in previous brucellaphage genomic studies. The first issue is the inconsistent naming of proteins with the same function. For example, ORF20 of Y17, after BLASTp comparison, shows over 99% identity and 100% coverage with ORF21 of Bk, Pr, R/C, S708, and Wb, indicating that they are likely the same protein. However, ORF21 is predicted to function as an amidase in Pr but as a structural protein in Bk, R/C, S708, and Wb. Similar discrepancies in predicted functions are observed across numerous other proteins (Supplementary Tables S1, S2). The functional proteins of Y17 were primarily named based on the detailed analysis of Pr and Tb genomes and proteins conducted by Flores et al. (8). Second, the circular nature of brucellaphage genomes may explain why the initiating proteins in some phages, such as Bk2, F1, Fi, and Iz, have yet to be correctly identified. Finally, genomic collinearity analysis revealed that many EF4 proteins appeared fragmented into multiple segments, likely due to genome assembly errors.

The prevention and control of brucellosis are highly intricate and challenging due to the high infectivity and pathogenicity of *Brucella*. Phage Y17 exhibited strong resistance to UV light, temperature, pH, and ethanol, indicating its exceptional adaptability to diverse environmental conditions. Moreover, no virulence or antibiotic resistance-associated genes were identified in the Y17 genome. Although Y17 was initially isolated using the *Brucella abortus* A19 vaccine strain, host range testing revealed its lytic activity against several clinical *B. melitensis* isolates. These characteristics make Y17 a promising candidate for the prevention and control of brucellosis.

## 5 Conclusion

In this study, we isolated and characterized a novel lytic brucellaphage, Y17, from Ludian County, Yunnan Province, contributing to the growing understanding of brucellaphages. Phage Y17 possesses the smallest genome and particle size among known brucellaphages. Compared to other brucellaphages, Y17 has a relatively shorter latent period and the largest burst size. Genome analysis confirmed its safety and potential for biocontrol applications against *Brucella*. This study expands our understanding of brucellaphages and offers a promising approach for environmental disinfection and the prevention of brucellosis in epidemic areas. However, the practical application of phages necessitates further investigation, including comprehensive validation of their antibacterial efficacy and the optimization of application strategies. Future research will focus on exploring functional phenotypes and medical applications to maximize their effectiveness in environmental disinfection and the treatment of brucellosis.

## Data Availability

The datasets presented in this study can be found in online repositories. The names of the repository/repositories and accession number(s) can be found in the article/Supplementary material.
