# Supplementary material for: Characteristics of the smallest brucellaphage with strong lytic ability
Source: Front Vet Sci. 2025 Feb 5;12:1530123. doi: 10.3389/fvets.2025.1530123 (PMC11836647; doi:10.3389/fvets.2025.1530123)
Supplement: Supplementary file 1 [file Data_Sheet_1.zip › Supplementary/Supplementary TableS 2.docx]

The general features of the best-matched gene products of phage Y17 from the balstp comparison with other lytic brucellaphages.

| ORF | bp | | strand | Annotated function | Highest homology Protein |
| --- | --- | --- | --- | --- | --- |
|  | start | stop |  |  |  |
| 1 | 274 | 471 | + | hypothetical proteint | hypothetical proteint 02_19(ORF1) |
|  |  |  |  |  | hypothetical protein 1066_19(ORF1) |
|  |  |  |  |  | hypothetical protein 110_19(ORF1) |
|  |  |  |  |  | hypothetical protein 11sa_19(ORF1) |
|  |  |  |  |  | hypothetical protein 141_19(ORF1) |
|  |  |  |  |  | hypothetical protein 177_19(ORF1) |
|  |  |  |  |  | hypothetical protein 281_19(ORF1) |
|  |  |  |  |  | hypothetical protein 544_19(ORF1) |
|  |  |  |  |  | hypothetical protein Bk(ORF1) |
|  |  |  |  |  | hypothetical protein Bk2(ORF54) |
|  |  |  |  |  | hypothetical protein EF4(CDS1) |
|  |  |  |  |  | hypothetical protein F1(ORF54) |
|  |  |  |  |  | hypothetical protein Fi(ORF54) |
|  |  |  |  |  | hypothetical protein Fz(ORF1) |
|  |  |  |  |  | hypothetical protein Iz(ORF61) |
|  |  |  |  |  | hypothetical protein Pr(ORF1) |
|  |  |  |  |  | hypothetical protein R/C(ORF1) |
|  |  |  |  |  | hypothetical protein S708(ORF1) |
|  |  |  |  |  | hypothetical protein Tb(ORF1) |
|  |  |  |  |  | hypothetical protein V_19(ORF1) |
|  |  |  |  |  | hypothetical protein Wb(ORF1) |
| 2 | 471 | 791 | + | hypothetical protein | hypothetical proteint 02_19(ORF2) |
|  |  |  |  |  | hypotheticaprotein 1066_19(ORF2) |
|  |  |  |  |  | hypothetical protein 110_19(ORF2) |
|  |  |  |  |  | hypothetical protein 11sa_19(ORF2) |
|  |  |  |  |  | hypothetical protein 141_19(ORF2) |
|  |  |  |  |  | hypothetical protein 177_19(ORF2) |
|  |  |  |  |  | hypothetical protein 281_19(ORF2) |
|  |  |  |  |  | hypothetical protein 544_19(ORF2) |
|  |  |  |  |  | hypothetical protein Bk(ORF2) |
|  |  |  |  |  | hypothetical protein Bk2(ORF55) |
|  |  |  |  |  | hypothetical protein EF4(CDS2) |
|  |  |  |  |  | hypothetical protein F1(ORF55) |
|  |  |  |  |  | hypothetical protein Fi(ORF55) |
|  |  |  |  |  | hypothetical protein Fz(ORF2) |
|  |  |  |  |  | hypothetical protein Iz(ORF62) |
|  |  |  |  |  | hypothetical protein Pr(ORF2) |
|  |  |  |  |  | hypothetical protein R/C(ORF2) |
|  |  |  |  |  | hypothetical protein S708(ORF2) |
|  |  |  |  |  | hypothetical protein Tb(ORF2) |
|  |  |  |  |  | hypothetical protein V_19(ORF2) |
|  |  |  |  |  | hypothetical protein Wb(ORF2) |
| 3 | 856 | 1020 | + | hypothetical protein | hypothetical proteint 02_19(ORF3) |
|  |  |  |  |  | hypothetical protein 1066_19(ORF3) |
|  |  |  |  |  | hypothetical protein 110_19(ORF3) |
|  |  |  |  |  | hypothetical protein 11sa_19(ORF3) |
|  |  |  |  |  | hypothetical protein 141_19(ORF3) |
|  |  |  |  |  | hypothetical protein 177_19(ORF3) |
|  |  |  |  |  | hypothetical protein 281_19(ORF3) |
|  |  |  |  |  | hypothetical protein 544_19(ORF3) |
|  |  |  |  |  | hypothetical protein Bk(ORF3) |
|  |  |  |  |  | hypothetical protein Bk2(ORF56) |
|  |  |  |  |  | hypothetical protein EF4(CDS3) |
|  |  |  |  |  | hypothetical protein F1(ORF56) |
|  |  |  |  |  | hypothetical protein Fi(ORF56) |
|  |  |  |  |  | hypothetical protein Fz(ORF3) |
|  |  |  |  |  | hypothetical protein Iz(ORF63) |
|  |  |  |  |  | hypothetical protein Pr(ORF3) |
|  |  |  |  |  | hypothetical protein R/C(ORF3) |
|  |  |  |  |  | hypothetical protein S708(ORF3) |
|  |  |  |  |  | hypothetical protein Tb(ORF3) |
|  |  |  |  |  | hypothetical protein V_19(ORF3) |
|  |  |  |  |  | hypothetical protein Wb(ORF3) |
| 4 | 1085 | 1762 | + | GcrA-like protein | GcrA-like proteint 02_19(ORF4) |
|  |  |  |  |  | GcrA-like protein 1066_19(ORF4) |
|  |  |  |  |  | GcrA-like protein 110_19(ORF4) |
|  |  |  |  |  | GcrA-like protein 11sa_19(ORF4) |
|  |  |  |  |  | GcrA-like protein 141_19(ORF4) |
|  |  |  |  |  | GcrA-like protein 177_19(ORF4) |
|  |  |  |  |  | GcrA-like protein 281_19(ORF4) |
|  |  |  |  |  | GcrA-like protein 544_19(ORF4) |
|  |  |  |  |  | GcrA-like protein Bk(ORF4) |
|  |  |  |  |  | cell cycle regulator Bk2(ORF57) |
|  |  |  |  |  | GcrA-like cell cycle regulator EF4(CDS4、5) |
|  |  |  |  |  | cell cycle regulator F1(ORF57) |
|  |  |  |  |  | cell cycle regulator Fi(ORF57) |
|  |  |  |  |  | GcrA-like protein Fz(ORF4) |
|  |  |  |  |  | cell cycle regulator Iz(ORF64) |
|  |  |  |  |  | GcrA-like protein Pr(ORF4) |
|  |  |  |  |  | GcrA-like protein R/C(ORF4) |
|  |  |  |  |  | GcrA-like protein S708(ORF4) |
|  |  |  |  |  | GcrA-like protein Tb(ORF4) |
|  |  |  |  |  | GcrA-like protein V_19(ORF4) |
|  |  |  |  |  | GcrA-like protein Wb(ORF4) |
| 5 | 1759 | 1851 | + | GcrA-like protein | hypothetical proteint 02_19(ORF5) |
|  |  |  |  |  | hypothetical protein 1066_19(ORF5) |
|  |  |  |  |  | hypothetical protein 110_19(ORF5) |
|  |  |  |  |  | GcrA-like protein 11sa_19(ORF5) |
|  |  |  |  |  | GcrA-like protein 141_19(ORF5) |
|  |  |  |  |  | hypothetical protein 177_19(ORF5) |
|  |  |  |  |  | hypothetical protein 281_19(ORF5) |
|  |  |  |  |  | hypothetical protein 544_19(ORF5) |
|  |  |  |  |  | hypothetical protein Bk(ORF5) |
|  |  |  |  |  | hypothetical protein Bk2(ORF58) |
|  |  |  |  |  | hypothetical protein F1(ORF58) |
|  |  |  |  |  | hypothetical protein Fi(ORF58) |
|  |  |  |  |  | GcrA-like protein Fz(ORF5) |
|  |  |  |  |  | hypothetical protein Pr(ORF5) |
|  |  |  |  |  | hypothetical protein R/C(ORF5) |
|  |  |  |  |  | hypothetical protein S708(ORF5) |
|  |  |  |  |  | hypothetical protein Tb(ORF5) |
|  |  |  |  |  | hypothetical protein V_19(ORF5) |
|  |  |  |  |  | hypothetical protein Wb(ORF5) |
| 6 | 1944 | 2342 | + | terminase small subunit | terminase small subunit 02_19(ORF6) |
|  |  |  |  |  | terminase small subunit 1066_19(ORF6) |
|  |  |  |  |  | terminase small subunit 110_19(ORF6) |
|  |  |  |  |  | terminase small subunit 11sa_19(ORF6) |
|  |  |  |  |  | terminase small subunit 141_19(ORF6) |
|  |  |  |  |  | terminase small subunit 177_19(ORF6) |
|  |  |  |  |  | terminase small subunit 281_19(ORF6) |
|  |  |  |  |  | terminase small subunit 544_19(ORF6) |
|  |  |  |  |  | terminase small subunit Bk(ORF6) |
|  |  |  |  |  | terminase small subunit Bk2(ORF1) |
|  |  |  |  |  | terminase small subunit EF4(CDS6、7) |
|  |  |  |  |  | terminase small subunit F1(ORF1) |
|  |  |  |  |  | terminase small subunit Fi(ORF1) |
|  |  |  |  |  | terminase small subunit Fz(ORF6) |
|  |  |  |  |  | terminase small subunit Iz(ORF1) |
|  |  |  |  |  | terminase small subunit Pr(ORF6) |
|  |  |  |  |  | terminase small subunit R/C(ORF6) |
|  |  |  |  |  | terminase small subunit S708(ORF6) |
|  |  |  |  |  | terminase small subunit Tb(ORF6) |
|  |  |  |  |  | terminase small subunit V_19(ORF6) |
|  |  |  |  |  | terminase small subunit Wb(ORF6) |
| 7 | 2347 | 4893 | + | terminase large subunit | terminase large subunit 02_19(ORF7) |
|  |  |  |  |  | terminase large subunit 1066_19(ORF7) |
|  |  |  |  |  | terminase large subunit 110_19(ORF7) |
|  |  |  |  |  | terminase large subunit 11sa_19(ORF7) |
|  |  |  |  |  | terminase large subunit 141_19(ORF7) |
|  |  |  |  |  | terminase large subunit 177_19(ORF7) |
|  |  |  |  |  | terminase large subunit 281_19(ORF7) |
|  |  |  |  |  | terminase large subunit 544_19(ORF7) |
|  |  |  |  |  | terminase large subunit BK(ORF7) |
|  |  |  |  |  | terminase large subunit Bk2(ORF2) |
|  |  |  |  |  | terminase large subunit EF4(CDS8、9、10) |
|  |  |  |  |  | terminase large subunit F1(ORF2) |
|  |  |  |  |  | terminase large subunit Fi(ORF2) |
|  |  |  |  |  | terminase large subunit Fz(ORF7) |
|  |  |  |  |  | terminase large subunit Iz(ORF2) |
|  |  |  |  |  | terminase large subunit Pr(ORF7) |
|  |  |  |  |  | terminase large subunit R/C(ORF7) |
|  |  |  |  |  | terminase large subunit S708(ORF7) |
|  |  |  |  |  | terminase large subunit Tb(ORF7) |
|  |  |  |  |  | terminase large subunit V_19(ORF7) |
|  |  |  |  |  | terminase large subunit Wb(ORF7) |
| 8 | 4943 | 5200 | + | hypothetical protein | hypothetical protein 02_19(ORF9) |
|  |  |  |  |  | hypothetical protein 1066_19(ORF9) |
|  |  |  |  |  | hypothetical protein 110_19(ORF9) |
|  |  |  |  |  | hypothetical protein 11sa_19(ORF9) |
|  |  |  |  |  | hypothetical protein 141_19(ORF9) |
|  |  |  |  |  | hypothetical protein 177_19(ORF9) |
|  |  |  |  |  | hypothetical protein 281_19(ORF9) |
|  |  |  |  |  | hypothetical protein 544_19(ORF9) |
|  |  |  |  |  | hypothetical protein Bk(ORF9) |
|  |  |  |  |  | hypothetical protein Bk2(ORF4) |
|  |  |  |  |  | hypothetical protein EF4(CDS11) |
|  |  |  |  |  | hypothetical protein F1(ORF4) |
|  |  |  |  |  | hypothetical protein Fi(ORF4) |
|  |  |  |  |  | hypothetical protein Fz(ORF9) |
|  |  |  |  |  | hypothetical protein Iz(ORF3) |
|  |  |  |  |  | hypothetical protein Pr(ORF9) |
|  |  |  |  |  | hypothetical protein R/C(ORF9) |
|  |  |  |  |  | hypothetical protein S708(ORF9) |
|  |  |  |  |  | hypothetical protein Tb(ORF9) |
|  |  |  |  |  | hypothetical protein V_19(ORF9) |
|  |  |  |  |  | hypothetical protein Wb(ORF9) |
| 9 | 5197 | 5490 | + | hypothetical protein | hypothetical protein 02_19(ORF10) |
|  |  |  |  |  | hypothetical protein 1066_19(ORF10) |
|  |  |  |  |  | hypothetical protein 110_19(ORF10) |
|  |  |  |  |  | hypothetical protein 11sa_19(ORF10) |
|  |  |  |  |  | hypothetical protein 141_19(ORF10) |
|  |  |  |  |  | hypothetical protein 177_19(ORF10) |
|  |  |  |  |  | hypothetical protein 281_19(ORF10) |
|  |  |  |  |  | hypothetical protein 544_19(ORF10) |
|  |  |  |  |  | hypothetical protein Bk(ORF19) |
|  |  |  |  |  | hypothetical protein Bk2(ORF5) |
|  |  |  |  |  | hypothetical protein EF4(CDS12) |
|  |  |  |  |  | hypothetical protein F1(ORF5) |
|  |  |  |  |  | hypothetical protein Fi(ORF5) |
|  |  |  |  |  | hypothetical protein Fz(ORF10) |
|  |  |  |  |  | hypothetical protein Iz(ORF4) |
|  |  |  |  |  | hypothetical protein Pr(ORF10) |
|  |  |  |  |  | hypothetical protein R/C(ORF10) |
|  |  |  |  |  | hypothetical protein S708(ORF10) |
|  |  |  |  |  | hypothetical protein Tb(ORF10) |
|  |  |  |  |  | hypothetical protein V_19(ORF10) |
|  |  |  |  |  | hypothetical protein Wb(ORF10) |
| 10 | 5490 | 5885 | + | hypothetical protein | hypothetical protein 02_19(ORF11) |
|  |  |  |  |  | hypothetical protein 1066_19(ORF11) |
|  |  |  |  |  | hypothetical protein 110_19(ORF11) |
|  |  |  |  |  | hypothetical protein 11sa_19(ORF11) |
|  |  |  |  |  | hypothetical protein 141_19(ORF11) |
|  |  |  |  |  | hypothetical protein 177_19(ORF11) |
|  |  |  |  |  | hypothetical protein 281_19(ORF11) |
|  |  |  |  |  | hypothetical protein 544_19(ORF11) |
|  |  |  |  |  | hypothetical protein Bk(ORF11) |
|  |  |  |  |  | hypothetical protein Bk2(ORF6) |
|  |  |  |  |  | hypothetical protein EF4(CDS13) |
|  |  |  |  |  | hypothetical protein F1(ORF6) |
|  |  |  |  |  | hypothetical protein Fi(ORF6) |
|  |  |  |  |  | hypothetical protein Fz(ORF11) |
|  |  |  |  |  | hypothetical protein Iz(ORF5) |
|  |  |  |  |  | hypothetical protein Pr(ORF11) |
|  |  |  |  |  | hypothetical protein R/C(ORF11) |
|  |  |  |  |  | hypothetical protein S708(ORF11) |
|  |  |  |  |  | hypothetical protein Tb(ORF11) |
|  |  |  |  |  | hypothetical protein V_19(ORF11) |
|  |  |  |  |  | hypothetical protein Wb(ORF11) |
| 11 | 6370 | 8694 | + | portal protein | portal protein 02_19(ORF12) |
|  |  |  |  |  | portal protein 1066_19(ORF12) |
|  |  |  |  |  | portal protein 110_19(ORF12) |
|  |  |  |  |  | portal protein 11sa_19(ORF12) |
|  |  |  |  |  | portal protein 141_19(ORF12) |
|  |  |  |  |  | portal protein 177_19(ORF12) |
|  |  |  |  |  | portal protein 281_19(ORF12) |
|  |  |  |  |  | portal protein 544_19(ORF12) |
|  |  |  |  |  | portal protein BK(ORF12) |
|  |  |  |  |  | portal protein Bk2(ORF7) |
|  |  |  |  |  | portal protein EF4(CDS14) |
|  |  |  |  |  | portal protein F1(ORF7) |
|  |  |  |  |  | portal protein Fi(ORF7) |
|  |  |  |  |  | portal protein Fz(ORF12) |
|  |  |  |  |  | virion structural protein Iz(ORF7) |
|  |  |  |  |  | portal protein Pr(ORF12) |
|  |  |  |  |  | portal protein R/C(ORF12) |
|  |  |  |  |  | portal protein S708(ORF12) |
|  |  |  |  |  | portal protein Tb(ORF12) |
|  |  |  |  |  | portal protein V_19(ORF12) |
|  |  |  |  |  | portal protein Wb(ORF12) |
| 12 | 8913 | 9092 | + | hypothetical protein | hypothetical protein EF4(CDS16) |
|  |  |  |  |  | hypothetical protein Iz(ORF9) |
| 13 | 9358 | 9176 | - | hypothetical protein | unique |
| 14 | 9551 | 9793 | + | hypothetical protein | hypothetical protein 02_19(ORF15) |
|  |  |  |  |  | hypothetical protein 1066_19(ORF15) |
|  |  |  |  |  | hypothetical protein 110_19(ORF15) |
|  |  |  |  |  | hypothetical protein 11sa_19(ORF15) |
|  |  |  |  |  | hypothetical protein 141_19(ORF15) |
|  |  |  |  |  | hypothetical protein 177_19(ORF15) |
|  |  |  |  |  | hypothetical protein 281_19(ORF15) |
|  |  |  |  |  | hypothetical protein 544_19(ORF15) |
|  |  |  |  |  | hypothetical protein Bk(ORF15) |
|  |  |  |  |  | hypothetical protein Bk2(ORF10) |
|  |  |  |  |  | hypothetical protein EF4(CDS18) |
|  |  |  |  |  | hypothetical protein F1(ORF10) |
|  |  |  |  |  | hypothetical protein Fi(ORF10) |
|  |  |  |  |  | hypothetical protein Fz(ORF15) |
|  |  |  |  |  | hypothetical protein Iz(ORF12) |
|  |  |  |  |  | hypothetical protein Pr(ORF15) |
|  |  |  |  |  | hypothetical protein R/C(ORF15) |
|  |  |  |  |  | hypothetical protein S708(ORF15) |
|  |  |  |  |  | hypothetical protein Tb(ORF15) |
|  |  |  |  |  | hypothetical protein V_19(ORF15) |
|  |  |  |  |  | hypothetical protein Wb(ORF15) |
| 15 | 9823 | 11004 | + | structural protein | structural protein 02_19(ORF16) |
|  |  |  |  |  | structural protein 1066_19(ORF16) |
|  |  |  |  |  | structural protein 110_19(ORF16) |
|  |  |  |  |  | structural protein 11sa_19(ORF16) |
|  |  |  |  |  | structural protein 141_19(ORF16) |
|  |  |  |  |  | structural protein 177_19(ORF16) |
|  |  |  |  |  | structural protein 281_19(ORF16) |
|  |  |  |  |  | structural protein 544_19(ORF16) |
|  |  |  |  |  | structural protein BK(ORF16) |
|  |  |  |  |  | hypothetical protein Bk2(ORF11) |
|  |  |  |  |  | structural protein EF4(CDS19) |
|  |  |  |  |  | structural protein F1(ORF11) |
|  |  |  |  |  | hypothetical protein Fi(ORF11) |
|  |  |  |  |  | structural protein Fz(ORF16) |
|  |  |  |  |  | hypothetical protein Iz(ORF13) |
|  |  |  |  |  | virion structural protein Pr(ORF16) |
|  |  |  |  |  | structural protein R/C(ORF16) |
|  |  |  |  |  | structural protein S708(ORF16) |
|  |  |  |  |  | virion structural protein Tb(ORF16) |
|  |  |  |  |  | structural protein V_19(ORF16) |
|  |  |  |  |  | structural protein Wb(ORF16) |
| 16 | 11014 | 12024 | + | head protein | major head protein 02_19(ORF17) |
|  |  |  |  |  | major head protein 1066_19(ORF17) |
|  |  |  |  |  | major head protein 110_19(ORF17) |
|  |  |  |  |  | major head protein 11sa_19(ORF17) |
|  |  |  |  |  | major head protein 141_19(ORF17) |
|  |  |  |  |  | major head protein 177_19(ORF17) |
|  |  |  |  |  | major head protein 281_19(ORF17) |
|  |  |  |  |  | major head protein 544_19(ORF17) |
|  |  |  |  |  | major head protein BK(ORF17) |
|  |  |  |  |  | major capsid protein Bk2(ORF12) |
|  |  |  |  |  | major head protein EF4(CDS20) |
|  |  |  |  |  | major capsid protein F1(ORF12) |
|  |  |  |  |  | major capsid protein Fi(ORF12) |
|  |  |  |  |  | major head protein Fz(ORF17) |
|  |  |  |  |  | major virion structural protein Iz(ORF14) |
|  |  |  |  |  | major head protein Pr(ORF17) |
|  |  |  |  |  | major head protein R/C(ORF17) |
|  |  |  |  |  | major head protein S708(ORF17) |
|  |  |  |  |  | major head protein Tb(ORF17) |
|  |  |  |  |  | major head protein V_19(ORF17) |
|  |  |  |  |  | major head protein Wb(ORF17) |
| 17 | 12037 | 12381 | + | structural protein | hypothetical protein 02_19(ORF18) |
|  |  |  |  |  | hypothetical protein 1066_19(ORF18) |
|  |  |  |  |  | hypothetical protein 110_19(ORF18) |
|  |  |  |  |  | hypothetical protein 11sa_19(ORF18) |
|  |  |  |  |  | hypothetical protein 141_19(ORF18) |
|  |  |  |  |  | hypothetical protein 177_19(ORF18) |
|  |  |  |  |  | hypothetical protein 281_19(ORF18) |
|  |  |  |  |  | hypothetical protein 544_19(ORF18) |
|  |  |  |  |  | structural protein BK(ORF18) |
|  |  |  |  |  | hypothetical protein Bk2(ORF13) |
|  |  |  |  |  | hypothetical protein EF4(CDS21) |
|  |  |  |  |  | hypothetical protein F1(ORF13) |
|  |  |  |  |  | hypothetical protein Fi(ORF13) |
|  |  |  |  |  | hypothetical protein Fz(ORF18) |
|  |  |  |  |  | hypothetical protein Iz(ORF15) |
|  |  |  |  |  | virion structural protein Pr(ORF18) |
|  |  |  |  |  | structural protein R/C(ORF18) |
|  |  |  |  |  | structural protein S708(ORF18) |
|  |  |  |  |  | virion structural protein Tb(ORF18) |
|  |  |  |  |  | hypothetical protein V_19(ORF18) |
|  |  |  |  |  | structural protein Wb(ORF18) |
| 18 | 12439 | 13122 | + | hypothetical protein | hypothetical protein 02_19(ORF19) |
|  |  |  |  |  | hypothetical protein 1066_19(ORF19) |
|  |  |  |  |  | hypothetical protein 110_19(ORF19) |
|  |  |  |  |  | hypothetical protein 11sa_19(ORF19) |
|  |  |  |  |  | hypothetical protein 141_19(ORF19) |
|  |  |  |  |  | hypothetical protein 177_19(ORF19) |
|  |  |  |  |  | hypothetical protein 281_19(ORF19) |
|  |  |  |  |  | hypothetical protein 544_19(ORF19) |
|  |  |  |  |  | hypothetical protein Bk(ORF19) |
|  |  |  |  |  | hypothetical protein Bk2(ORF14) |
|  |  |  |  |  | hypothetical protein EF4(CDS22) |
|  |  |  |  |  | hypothetical protein F1(ORF14) |
|  |  |  |  |  | hypothetical protein Fi(ORF14) |
|  |  |  |  |  | hypothetical protein Fz(ORF19) |
|  |  |  |  |  | hypothetical protein Iz(ORF16) |
|  |  |  |  |  | hypothetical protein Pr(ORF19) |
|  |  |  |  |  | hypothetical protein R/C(ORF19) |
|  |  |  |  |  | hypothetical protein S708(ORF19) |
|  |  |  |  |  | hypothetical protein Tb(ORF19) |
|  |  |  |  |  | hypothetical protein V_19(ORF19) |
|  |  |  |  |  | hypothetical protein Wb(ORF19) |
| 19 | 13123 | 13824 | + | tail protein | structural protein 02_19(ORF20) |
|  |  |  |  |  | structural protein 1066_19(ORF20) |
|  |  |  |  |  | structural protein 110_19(ORF20) |
|  |  |  |  |  | structural protein 11sa_19(ORF20) |
|  |  |  |  |  | structural protein 141_19(ORF20) |
|  |  |  |  |  | structural protein 177_19(ORF20) |
|  |  |  |  |  | structural protein 281_19(ORF20) |
|  |  |  |  |  | structural protein 544_19(ORF20) |
|  |  |  |  |  | structural protein BK(ORF20) |
|  |  |  |  |  | hypothetical protein Bk2(ORF15) |
|  |  |  |  |  | structural protein EF4(CDS23、24) |
|  |  |  |  |  | structural protein F1(ORF15) |
|  |  |  |  |  | hypothetical protein Fi(ORF15) |
|  |  |  |  |  | structural protein Fz(ORF20) |
|  |  |  |  |  | hypothetical protein Iz(ORF17) |
|  |  |  |  |  | tail protein Pr(ORF20) |
|  |  |  |  |  | structural protein R/C(ORF20) |
|  |  |  |  |  | structural protein S708(ORF20) |
|  |  |  |  |  | tail protein Tb(ORF20) |
|  |  |  |  |  | structural protein V_19(ORF20) |
|  |  |  |  |  | structural protein Wb(ORF20) |
| 20 | 13828 | 15765 | + | amidase | structural protein 02_19(ORF21) |
|  |  |  |  |  | structural protein 1066_19(ORF21) |
|  |  |  |  |  | structural protein 110_19(ORF21) |
|  |  |  |  |  | structural protein 11sa_19(ORF21) |
|  |  |  |  |  | structural protein 141_19(ORF21) |
|  |  |  |  |  | structural protein 177_19(ORF21) |
|  |  |  |  |  | structural protein 281_19(ORF21) |
|  |  |  |  |  | structural protein 544_19(ORF21) |
|  |  |  |  |  | structural protein BK(ORF21) |
|  |  |  |  |  | hypothetical protein Bk2(ORF16) |
|  |  |  |  |  | structural protein EF4(CDS25、26、27、28) |
|  |  |  |  |  | structural protein F1(ORF16) |
|  |  |  |  |  | hypothetical protein Fi(ORF16) |
|  |  |  |  |  | structural protein Fz(ORF21) |
|  |  |  |  |  | neck structural protein Iz(ORF18) |
|  |  |  |  |  | amidase Pr(ORF21) |
|  |  |  |  |  | structural protein R/C(ORF21) |
|  |  |  |  |  | structural protein S708(ORF21) |
|  |  |  |  |  | amidase Tb(ORF21) |
|  |  |  |  |  | structural protein V_19(ORF21) |
|  |  |  |  |  | structural protein Wb(ORF21) |
| 21 | 15774 | 16214 | + | acyl-CoA N-acyltransferase | hypothetical protein 02_19(ORF22) |
|  |  |  |  |  | hypothetical protein 1066_19(ORF22) |
|  |  |  |  |  | hypothetical protein 110_19(ORF22) |
|  |  |  |  |  | hypothetical protein 11sa_19(ORF22) |
|  |  |  |  |  | hypothetical protein 141_19(ORF22) |
|  |  |  |  |  | hypothetical protein 177_19(ORF22) |
|  |  |  |  |  | hypothetical protein 281_19(ORF22) |
|  |  |  |  |  | hypothetical protein 544_19(ORF22) |
|  |  |  |  |  | hypothetical protein Bk(ORF22) |
|  |  |  |  |  | hypothetical protein Bk2(ORF17) |
|  |  |  |  |  | N-acetyltransferase EF4(CDS29) |
|  |  |  |  |  | hypothetical protein F1(ORF17) |
|  |  |  |  |  | hypothetical protein Fi(ORF17) |
|  |  |  |  |  | hypothetical protein Fz(ORF22) |
|  |  |  |  |  | acetyltransferase Iz(ORF19) |
|  |  |  |  |  | acyl-CoA N-acyltransferase Pr(ORF22) |
|  |  |  |  |  | hypothetical protein R/C(ORF22) |
|  |  |  |  |  | hypothetical protein S708(ORF22) |
|  |  |  |  |  | acyl-CoA N-acyltransferase Tb(ORF22) |
|  |  |  |  |  | hypothetical protein V_19(ORF22) |
|  |  |  |  |  | hypothetical protein Wb(ORF22) |
| 22 | 16214 | 17194 | + | structural protein | structural protein 02_19(ORF23) |
|  |  |  |  |  | structural protein 1066_19(ORF23) |
|  |  |  |  |  | structural protein 110_19(ORF23) |
|  |  |  |  |  | structural protein 11sa_19(ORF23) |
|  |  |  |  |  | structural protein 141_19(ORF23) |
|  |  |  |  |  | structural protein 177_19(ORF23) |
|  |  |  |  |  | structural protein 281_19(ORF23) |
|  |  |  |  |  | structural protein 544_19(ORF23) |
|  |  |  |  |  | structural protein BK(ORF23) |
|  |  |  |  |  | hypothetical protein Bk2(ORF18) |
|  |  |  |  |  | hypothetical protein EF4(CDS30、31) |
|  |  |  |  |  | structural protein F1(ORF18) |
|  |  |  |  |  | hypothetical protein Fi(ORF18) |
|  |  |  |  |  | structural protein Fz(ORF23) |
|  |  |  |  |  | hypothetical protein Iz(ORF20) |
|  |  |  |  |  | virion structural protein Pr(ORF23) |
|  |  |  |  |  | structural protein R/C(ORF23) |
|  |  |  |  |  | structural protein S708(ORF23) |
|  |  |  |  |  | virion structural protein Tb(ORF23) |
|  |  |  |  |  | structural protein V_19(ORF23) |
|  |  |  |  |  | structural protein Wb(ORF23) |
| 23 | 17194 | 17610 | + | hypothetical protein | hypothetical protein 02_19(ORF24) |
|  |  |  |  |  | hypothetical protein 1066_19(ORF24) |
|  |  |  |  |  | hypothetical protein 110_19(ORF24) |
|  |  |  |  |  | hypothetical protein 11sa_19(ORF24) |
|  |  |  |  |  | hypothetical protein 141_19(ORF24) |
|  |  |  |  |  | hypothetical protein 177_19(ORF24) |
|  |  |  |  |  | hypothetical protein 281_19(ORF24) |
|  |  |  |  |  | hypothetical protein 544_19(ORF24) |
|  |  |  |  |  | hypothetical protein Bk(ORF24) |
|  |  |  |  |  | hypothetical protein Bk2(ORF19) |
|  |  |  |  |  | hypothetical protein EF4(CDS32) |
|  |  |  |  |  | hypothetical protein F1(ORF19) |
|  |  |  |  |  | hypothetical protein Fi(ORF19) |
|  |  |  |  |  | hypothetical protein Fz(ORF24) |
|  |  |  |  |  | hypothetical protein Iz(ORF21) |
|  |  |  |  |  | hypothetical protein Pr(ORF24) |
|  |  |  |  |  | hypothetical protein R/C(ORF24) |
|  |  |  |  |  | hypothetical protein S708(ORF24) |
|  |  |  |  |  | hypothetical protein Tb(ORF24) |
|  |  |  |  |  | hypothetical protein V_19(ORF24) |
|  |  |  |  |  | hypothetical protein Wb(ORF24) |
| 24 | 17610 | 19799 | + | peptidoglycan hydrolase | peptidoglycan hydrolase 02_19(ORF25) |
|  |  |  |  |  | Peptidoglycan hydrolase 1066_19(ORF25) |
|  |  |  |  |  | peptidoglycan hydrolase 110_19(ORF25) |
|  |  |  |  |  | peptidoglycan hydrolase 11sa_19(ORF25) |
|  |  |  |  |  | Peptidoglycan hydrolase 141_19(ORF25) |
|  |  |  |  |  | Peptidoglycan hydrolase 177_19(ORF25) |
|  |  |  |  |  | peptidoglycan hydrolase 281_19(ORF25) |
|  |  |  |  |  | peptidoglycan hydrolase 544_19(ORF25) |
|  |  |  |  |  | peptidoglycan hydrolase BK(ORF25) |
|  |  |  |  |  | peptidoglycan hydrolase Bk2(ORF20) |
|  |  |  |  |  | peptidoglycan hydrolase EF4(CDS32、33、34) |
|  |  |  |  |  | peptidoglycan hydrolase F1(ORF20) |
|  |  |  |  |  | peptidoglycan hydrolase Fi(ORF20) |
|  |  |  |  |  | peptidoglycan hydrolase Fz(ORF25) |
|  |  |  |  |  | peptidogylcan hydrolase Iz(ORF22) |
|  |  |  |  |  | peptidoglycan hydrolase Pr(ORF25) |
|  |  |  |  |  | peptidoglycan hydrolase R/C(ORF25) |
|  |  |  |  |  | peptidoglycan hydrolase S708(ORF25) |
|  |  |  |  |  | peptidoglycan hydrolase Tb(ORF25) |
|  |  |  |  |  | peptidoglycan hydrolase V_19(ORF25) |
|  |  |  |  |  | peptidoglycan hydrolase Wb(ORF25) |
| 25 | 19799 | 21301 | + | head protein | structural protein 02_19(ORF26) |
|  |  |  |  |  | structural protein 1066_19(ORF26) |
|  |  |  |  |  | structural protein 110_19(ORF26) |
|  |  |  |  |  | structural protein 11sa_19(ORF26) |
|  |  |  |  |  | structural protein 141_19(ORF26) |
|  |  |  |  |  | structural protein 177_19(ORF26) |
|  |  |  |  |  | structural protein 281_19(ORF26) |
|  |  |  |  |  | structural protein 544_19(ORF26) |
|  |  |  |  |  | structural protein BK(ORF26) |
|  |  |  |  |  | hypothetical protein Bk2(ORF21) |
|  |  |  |  |  | structural protein EF4(CDS35、36、37、38) |
|  |  |  |  |  | structural protein F1(ORF21) |
|  |  |  |  |  | hypothetical protein Fi(ORF21) |
|  |  |  |  |  | structural protein Fz(ORF26) |
|  |  |  |  |  | hypothetical protein Iz(ORF23) |
|  |  |  |  |  | major head protein Pr(ORF26) |
|  |  |  |  |  | structural protein R/C(ORF26) |
|  |  |  |  |  | structural protein S708(ORF26) |
|  |  |  |  |  | major head protein Tb(ORF26) |
|  |  |  |  |  | structural protein V_19(ORF26) |
|  |  |  |  |  | structural protein Wb(ORF26) |
| 26 | 21303 | 22160 | + | tail collar protein | tail collar protein 02_19(ORF27) |
|  |  |  |  |  | tail collar protein 1066_19(ORF27) |
|  |  |  |  |  | tail collar protein 110_19(ORF27) |
|  |  |  |  |  | tail collar protein 11sa_19(ORF27) |
|  |  |  |  |  | tail collar protein 141_19(ORF27) |
|  |  |  |  |  | tail collar protein 177_19(ORF27) |
|  |  |  |  |  | tail collar protein 281_19(ORF27) |
|  |  |  |  |  | tail collar protein 544_19(ORF27) |
|  |  |  |  |  | tail collar protein BK(ORF27) |
|  |  |  |  |  | tail collar protein Bk2(ORF22) |
|  |  |  |  |  | tail collar protein EF4(CDS39、40) |
|  |  |  |  |  | tail collar protein F1(ORF22) |
|  |  |  |  |  | tail collar protein Fi(ORF22) |
|  |  |  |  |  | tail collar protein Fz(ORF27) |
|  |  |  |  |  | tail collar domain-containing protein Iz(ORF24) |
|  |  |  |  |  | tail protein Pr(ORF27) |
|  |  |  |  |  | tail collar protein R/C(ORF27) |
|  |  |  |  |  | tail collar protein S708(ORF27) |
|  |  |  |  |  | tail protein Tb(ORF27) |
|  |  |  |  |  | tail collar protein V_19(ORF27) |
|  |  |  |  |  | tail collar protein Wb(ORF27) |
| 27 | 22160 | 22309 | + | hypothetical protein | hypothetical protein 02_19(ORF28) |
|  |  |  |  |  | hypothetical protein 1066_19(ORF28) |
|  |  |  |  |  | hypothetical protein 110_19(ORF28) |
|  |  |  |  |  | hypothetical protein 11sa_19(ORF28) |
|  |  |  |  |  | hypothetical protein 141_19(ORF28) |
|  |  |  |  |  | hypothetical protein 177_19(ORF28) |
|  |  |  |  |  | hypothetical protein 281_19(ORF28) |
|  |  |  |  |  | hypothetical protein 544_19(ORF28) |
|  |  |  |  |  | hypothetical protein Bk(ORF28) |
|  |  |  |  |  | hypothetical protein Bk2(ORF23) |
|  |  |  |  |  | hypothetical protein EF4(CDS41) |
|  |  |  |  |  | hypothetical protein F1(ORF23) |
|  |  |  |  |  | hypothetical protein Fi(ORF23) |
|  |  |  |  |  | hypothetical protein Fz(ORF28) |
|  |  |  |  |  | hypothetical protein Iz(ORF25) tail fiber protein Iz(ORF26) |
|  |  |  |  |  | hypothetical protein Pr(ORF28) |
|  |  |  |  |  | hypothetical protein R/C(ORF28) |
|  |  |  |  |  | hypothetical protein S708(ORF28) |
|  |  |  |  |  | hypothetical protein Tb(ORF28) |
|  |  |  |  |  | hypothetical protein V_19(ORF28) |
|  |  |  |  |  | hypothetical protein Wb(ORF28) |
| 28 | 22299 | 23114 | + | endolysin | endolysin 02_19(ORF30) |
|  |  |  |  |  | endolysin 1066_19(ORF30) |
|  |  |  |  |  | endolysin 110_19(ORF30) |
|  |  |  |  |  | endolysin 11sa_19(ORF30) |
|  |  |  |  |  | endolysin 141_19(ORF30) |
|  |  |  |  |  | endolysin 177_19(ORF30) |
|  |  |  |  |  | endolysin 281_19(ORF30) |
|  |  |  |  |  | endolysin 544_19(ORF30) |
|  |  |  |  |  | endolysin BK(ORF29) |
|  |  |  |  |  | endolysin Bk2(ORF24) |
|  |  |  |  |  | endolysin EF4(CDS42) |
|  |  |  |  |  | endolysin F1(ORF25) |
|  |  |  |  |  | endolysin Fi(ORF25) |
|  |  |  |  |  | endolysin Fz(ORF30) |
|  |  |  |  |  | secretion activator Iz(ORF28) |
|  |  |  |  |  | endolysin Pr(ORF29) |
|  |  |  |  |  | endolysin R/C(ORF29) |
|  |  |  |  |  | endolysin S708(ORF29) |
|  |  |  |  |  | endolysin Tb(ORF30) |
|  |  |  |  |  | endolysin V_19(ORF30) |
|  |  |  |  |  | endolysin Wb(ORF29) |
| 29 | 23107 | 23316 | + | hypothetical protein | hypothetical protein 02_19(ORF31) |
|  |  |  |  |  | hypothetical protein 1066_19(ORF31) |
|  |  |  |  |  | hypothetical protein 110_19(ORF31) |
|  |  |  |  |  | hypothetical protein 11sa_19(ORF31) |
|  |  |  |  |  | hypothetical protein 141_19(ORF31) |
|  |  |  |  |  | hypothetical protein 177_19(ORF31) |
|  |  |  |  |  | hypothetical protein 281_19(ORF31) |
|  |  |  |  |  | hypothetical protein 544_19(ORF31) |
|  |  |  |  |  | hypothetical protein Bk(ORF30) |
|  |  |  |  |  | hypothetical protein Bk2(ORF25) |
|  |  |  |  |  | hypothetical protein EF4(CDS43) |
|  |  |  |  |  | hypothetical protein F1(ORF26) |
|  |  |  |  |  | hypothetical protein Fi(ORF26) |
|  |  |  |  |  | hypothetical protein Fz(ORF31) |
|  |  |  |  |  | hypothetical protein Iz(ORF29) |
|  |  |  |  |  | hypothetical protein Pr(ORF30) |
|  |  |  |  |  | hypothetical protein R/C(ORF30) |
|  |  |  |  |  | hypothetical protein S708(ORF30) |
|  |  |  |  |  | hypothetical protein Tb(ORF31) |
|  |  |  |  |  | hypothetical protein V_19(ORF31) |
|  |  |  |  |  | hypothetical protein Wb(ORF30) |
| 30 | 23452 | 23859 | + | hypothetical protein | hypothetical protein Pr(ORF31) |
|  |  |  |  |  | hypothetical protein R/C(ORF31) |
|  |  |  |  |  | hypothetical protein S708(ORF31) |
|  |  |  |  |  | hypothetical protein Tb(ORF32) |
|  |  |  |  |  | hypothetical protein V_19(ORF32) |
|  |  |  |  |  | hypothetical protein Wb(ORF31) |
|  |  |  |  |  | hypothetical protein 281_19(ORF32) |
|  |  |  |  |  | hypothetical protein 544_19(ORF32) |
|  |  |  |  |  | hypothetical protein Bk(ORF31) |
|  |  |  |  |  | hypothetical protein Bk2(ORF26) |
|  |  |  |  |  | hypothetical protein EF4(CDS44) |
|  |  |  |  |  | hypothetical protein F1(ORF27) |
|  |  |  |  |  | hypothetical protein Fi(ORF27) |
|  |  |  |  |  | hypothetical protein Fz(ORF32) |
|  |  |  |  |  | hypothetical protein Pr(ORF31) |
|  |  |  |  |  | hypothetical protein R/C(ORF31) |
|  |  |  |  |  | hypothetical protein S708(ORF31) |
|  |  |  |  |  | hypothetical protein Tb(ORF32) |
|  |  |  |  |  | hypothetical protein V_19(ORF32) |
|  |  |  |  |  | hypothetical protein Wb(ORF31) |
| 31 | 24325 | 24062 | _ | hypothetical protein | hypothetical protein 02_19(ORF34) |
|  |  |  |  |  | hypothetical protein 1066_19(ORF34) |
|  |  |  |  |  | hypothetical protein 110_19(ORF34) |
|  |  |  |  |  | hypothetical protein 11sa_19(ORF34) |
|  |  |  |  |  | hypothetical protein 141_19(ORF34) |
|  |  |  |  |  | hypothetical protein 177_19(ORF34) |
|  |  |  |  |  | hypothetical protein 281_19(ORF34) |
|  |  |  |  |  | hypothetical protein 544_19(ORF34) |
|  |  |  |  |  | hypothetical protein Bk(ORF33) |
|  |  |  |  |  | hypothetical protein Bk2(ORF28) |
|  |  |  |  |  | hypothetical protein EF4(CDS46) |
|  |  |  |  |  | hypothetical protein F1(ORF29) |
|  |  |  |  |  | hypothetical protein Fi(ORF29) |
|  |  |  |  |  | hypothetical protein Fz(ORF34) |
|  |  |  |  |  | hypothetical protein Iz(ORF32) |
|  |  |  |  |  | hypothetical protein Pr(ORF33) |
|  |  |  |  |  | hypothetical protein R/C(ORF33) |
|  |  |  |  |  | hypothetical protein S708(ORF33) |
|  |  |  |  |  | hypothetical protein Tb(ORF34) |
|  |  |  |  |  | hypothetical protein V_19(ORF34) |
|  |  |  |  |  | hypothetical protein Wb(ORF33) |
| 32 | 24540 | 24328 | _ | hypothetical protein | hypothetical protein 02_19(ORF35) |
|  |  |  |  |  | hypothetical protein 1066_19(ORF35) |
|  |  |  |  |  | hypothetical protein 110_19(ORF35) |
|  |  |  |  |  | hypothetical protein 11sa_19(ORF35) |
|  |  |  |  |  | hypothetical protein 141_19(ORF35) |
|  |  |  |  |  | hypothetical protein 177_19(ORF35) |
|  |  |  |  |  | hypothetical protein 281_19(ORF35) |
|  |  |  |  |  | hypothetical protein 544_19(ORF35) |
|  |  |  |  |  | hypothetical protein Bk(ORF34) |
|  |  |  |  |  | hypothetical protein Bk2(ORF29) |
|  |  |  |  |  | hypothetical protein EF4(CDS47) |
|  |  |  |  |  | hypothetical protein F1(ORF30) |
|  |  |  |  |  | hypothetical protein Fi(ORF30) |
|  |  |  |  |  | hypothetical protein Fz(ORF35) |
|  |  |  |  |  | hypothetical protein Iz(ORF33) |
|  |  |  |  |  | hypothetical protein Pr(ORF34) |
|  |  |  |  |  | hypothetical protein R/C(ORF34) |
|  |  |  |  |  | hypothetical protein S708(ORF34) |
|  |  |  |  |  | hypothetical protein Tb(ORF35) |
|  |  |  |  |  | hypothetical protein V_19(ORF35) |
|  |  |  |  |  | hypothetical protein Wb(ORF34) |
| 33 | 24763 | 24533 | _ | hypothetical protein | hypothetical protein 02_19(ORF36) |
|  |  |  |  |  | hypothetical protein 1066_19(ORF36) |
|  |  |  |  |  | hypothetical protein 110_19(ORF36) |
|  |  |  |  |  | hypothetical protein 11sa_19(ORF36) |
|  |  |  |  |  | hypothetical protein 141_19(ORF36) |
|  |  |  |  |  | hypothetical protein 177_19(ORF36) |
|  |  |  |  |  | hypothetical protein 281_19(ORF36) |
|  |  |  |  |  | hypothetical protein 544_19(ORF36) |
|  |  |  |  |  | hypothetical protein Bk(ORF35) |
|  |  |  |  |  | hypothetical protein Bk2(ORF30) |
|  |  |  |  |  | hypothetical protein EF4(CDS48) |
|  |  |  |  |  | hypothetical protein F1(ORF31) |
|  |  |  |  |  | hypothetical protein Fi(ORF31) |
|  |  |  |  |  | hypothetical protein Fz(ORF36) |
|  |  |  |  |  | hypothetical protein Iz(ORF34) |
|  |  |  |  |  | hypothetical protein Pr(ORF35) |
|  |  |  |  |  | hypothetical protein R/C(ORF35) |
|  |  |  |  |  | hypothetical protein S708(ORF35) |
|  |  |  |  |  | hypothetical protein Tb(ORF36) |
|  |  |  |  |  | hypothetical protein V_19(ORF36) |
|  |  |  |  |  | hypothetical protein Wb(ORF35) |
| 34 | 25074 | 24760 | _ | hypothetical protein | hypothetical protein 02_19(ORF37) |
|  |  |  |  |  | hypothetical protein 1066_19(ORF37) |
|  |  |  |  |  | hypothetical protein 110_19(ORF37) |
|  |  |  |  |  | hypothetical protein 11sa_19(ORF37) |
|  |  |  |  |  | hypothetical protein 141_19(ORF37) |
|  |  |  |  |  | hypothetical protein 177_19(ORF37) |
|  |  |  |  |  | hypothetical protein 281_19(ORF37) |
|  |  |  |  |  | hypothetical protein 544_19(ORF37) |
|  |  |  |  |  | hypothetical protein Bk(ORF36) |
|  |  |  |  |  | hypothetical protein Bk2(ORF31) |
|  |  |  |  |  | hypothetical protein EF4(CDS48) |
|  |  |  |  |  | hypothetical protein F1(ORF32) |
|  |  |  |  |  | hypothetical protein Fi(ORF32) |
|  |  |  |  |  | hypothetical protein Fz(ORF37) |
|  |  |  |  |  | hypothetical protein Iz(ORF35) |
|  |  |  |  |  | hypothetical protein Pr(ORF36) |
|  |  |  |  |  | hypothetical protein R/C(ORF36) |
|  |  |  |  |  | hypothetical protein S708(ORF36) |
|  |  |  |  |  | hypothetical protein Tb(ORF37) |
|  |  |  |  |  | hypothetical protein V_19(ORF37) |
|  |  |  |  |  | hypothetical protein Wb(ORF36) |
| 35 | 25375 | 25064 | _ | hypothetical protein | hypothetical protein 02_19(ORF38) |
|  |  |  |  |  | hypothetical protein 1066_19(ORF38) |
|  |  |  |  |  | hypothetical protein 110_19(ORF38) |
|  |  |  |  |  | hypothetical protein 11sa_19(ORF38) |
|  |  |  |  |  | hypothetical protein 141_19(ORF38) |
|  |  |  |  |  | hypothetical protein 177_19(ORF38) |
|  |  |  |  |  | hypothetical protein 281_19(ORF38) |
|  |  |  |  |  | hypothetical protein 544_19(ORF38) |
|  |  |  |  |  | hypothetical protein Bk(ORF37) |
|  |  |  |  |  | hypothetical protein Bk2(ORF32) |
|  |  |  |  |  | hypothetical protein EF4(CDS49) |
|  |  |  |  |  | hypothetical protein F1(ORF33) |
|  |  |  |  |  | hypothetical protein Fi(ORF33) |
|  |  |  |  |  | hypothetical protein Fz(ORF38) |
|  |  |  |  |  | hypothetical protein Iz(ORF36) |
|  |  |  |  |  | hypothetical protein Pr(ORF37) |
|  |  |  |  |  | hypothetical protein R/C(ORF37) |
|  |  |  |  |  | hypothetical protein S708(ORF37) |
|  |  |  |  |  | hypothetical protein Tb(ORF38) |
|  |  |  |  |  | hypothetical protein V_19(ORF38) |
|  |  |  |  |  | hypothetical protein Wb(ORF37) |
| 36 | 25487 | 25368 | _ | hypothetical protein | hypothetical protein 02_19(ORF39) |
|  |  |  |  |  | hypothetical protein 1066_19(ORF39) |
|  |  |  |  |  | hypothetical protein 110_19(ORF39) |
|  |  |  |  |  | hypothetical protein 11sa_19(ORF39) |
|  |  |  |  |  | hypothetical protein 141_19(ORF39) |
|  |  |  |  |  | hypothetical protein 177_19(ORF39) |
|  |  |  |  |  | hypothetical protein 281_19(ORF39) |
|  |  |  |  |  | hypothetical protein 544_19(ORF39) |
|  |  |  |  |  | hypothetical protein Bk(ORF38) |
|  |  |  |  |  | hypothetical protein Bk2(ORF33) |
|  |  |  |  |  | hypothetical protein EF4(CDS50) |
|  |  |  |  |  | hypothetical protein F1(ORF34) |
|  |  |  |  |  | hypothetical protein Fi(ORF34) |
|  |  |  |  |  | hypothetical protein Fz(ORF39) |
|  |  |  |  |  | hypothetical protein Iz(ORF37) |
|  |  |  |  |  | hypothetical protein Pr(ORF38) |
|  |  |  |  |  | hypothetical protein R/C(ORF38) |
|  |  |  |  |  | hypothetical protein S708(ORF38) |
|  |  |  |  |  | hypothetical protein Tb(ORF39) |
|  |  |  |  |  | hypothetical protein V_19(ORF39) |
|  |  |  |  |  | hypothetical protein Wb(ORF38) |
| 37 | 25671 | 25522 | _ | hypothetical protein | hypothetical protein 02_19(ORF40) |
|  |  |  |  |  | hypothetical protein 1066_19(ORF40) |
|  |  |  |  |  | hypothetical protein 110_19(ORF40) |
|  |  |  |  |  | hypothetical protein 11sa_19(ORF40) |
|  |  |  |  |  | hypothetical protein 141_19(ORF40) |
|  |  |  |  |  | hypothetical protein 177_19(ORF40) |
|  |  |  |  |  | hypothetical protein 281_19(ORF40) |
|  |  |  |  |  | hypothetical protein 544_19(ORF40) |
|  |  |  |  |  | hypothetical protein Bk(ORF39) |
|  |  |  |  |  | hypothetical protein Bk2(ORF34) |
|  |  |  |  |  | hypothetical protein EF4(CDS51) |
|  |  |  |  |  | hypothetical protein F1(ORF35) |
|  |  |  |  |  | hypothetical protein Fi(ORF35) |
|  |  |  |  |  | hypothetical protein Fz(ORF40) |
|  |  |  |  |  | hypothetical protein Iz(ORF38) |
|  |  |  |  |  | hypothetical protein Pr(ORF39) |
|  |  |  |  |  | hypothetical protein R/C(ORF39) |
|  |  |  |  |  | hypothetical protein S708(ORF39) |
|  |  |  |  |  | hypothetical protein Tb(ORF40) |
|  |  |  |  |  | hypothetical protein V_19(ORF40) |
|  |  |  |  |  | hypothetical protein Wb(ORF39) |
| 38 | 25835 | 25668 | _ | hypothetical protein | hypothetical protein 02_19(ORF41) |
|  |  |  |  |  | hypothetical protein 1066_19(ORF41) |
|  |  |  |  |  | hypothetical protein 110_19(ORF41) |
|  |  |  |  |  | hypothetical protein 11sa_19(ORF41) |
|  |  |  |  |  | hypothetical protein 141_19(ORF41) |
|  |  |  |  |  | hypothetical protein 177_19(ORF41) |
|  |  |  |  |  | hypothetical protein 281_19(ORF41) |
|  |  |  |  |  | hypothetical protein 544_19(ORF41) |
|  |  |  |  |  | hypothetical protein Bk(ORF40) |
|  |  |  |  |  | hypothetical protein Bk2(ORF35) |
|  |  |  |  |  | hypothetical protein EF4(CDS51) |
|  |  |  |  |  | hypothetical protein F1(ORF36) |
|  |  |  |  |  | hypothetical protein Fi(ORF36) |
|  |  |  |  |  | hypothetical protein Fz(ORF41) |
|  |  |  |  |  | hypothetical protein Iz(ORF39) |
|  |  |  |  |  | hypothetical protein Pr(ORF40) |
|  |  |  |  |  | hypothetical protein R/C(ORF40) |
|  |  |  |  |  | hypothetical protein S708(ORF40) |
|  |  |  |  |  | hypothetical protein Tb(ORF41) |
|  |  |  |  |  | hypothetical protein V_19(ORF41) |
|  |  |  |  |  | hypothetical protein Wb(ORF40) |
| 39 | 26788 | 25832 | _ | hypothetical protein | hypothetical protein 02_19(ORF42) |
|  |  |  |  |  | hypothetical protein 1066_19(ORF42) |
|  |  |  |  |  | hypothetical protein 110_19(ORF42) |
|  |  |  |  |  | hypothetical protein 11sa_19(ORF42) |
|  |  |  |  |  | hypothetical protein 141_19(ORF42) |
|  |  |  |  |  | hypothetical protein 177_19(ORF42) |
|  |  |  |  |  | hypothetical protein 281_19(ORF42) |
|  |  |  |  |  | hypothetical protein 544_19(ORF42) |
|  |  |  |  |  | hypothetical protein Bk(ORF41) |
|  |  |  |  |  | hypothetical protein Bk2(ORF36) |
|  |  |  |  |  | hypothetical protein EF4(CDS52、53) |
|  |  |  |  |  | hypothetical protein F1(ORF37) |
|  |  |  |  |  | hypothetical protein Fi(ORF37) |
|  |  |  |  |  | hypothetical protein Fz(ORF42) |
|  |  |  |  |  | hypothetical protein Iz(ORF40) |
|  |  |  |  |  | hypothetical protein Pr(ORF41) |
|  |  |  |  |  | hypothetical protein R/C(ORF41) |
|  |  |  |  |  | hypothetical protein S708(ORF41) |
|  |  |  |  |  | hypothetical protein Tb(ORF42) |
|  |  |  |  |  | hypothetical protein V_19(ORF42) |
|  |  |  |  |  | hypothetical protein Wb(ORF41) |
| 40 | 26966  27605 | 26778  27018 | _  _ | DNA methyltransferase | DNA methyltransferase 02_19(ORF43) |
|  |  |  |  |  | DNA methyltransferase 1066_19(ORF43) |
|  |  |  |  |  | DNA methyltransferase 110_19(ORF43) |
|  |  |  |  |  | DNA methyltransferase 11sa_19(ORF43) |
|  |  |  |  |  | DNA methyltransferase 141_19(ORF43) |
|  |  |  |  |  | DNA methyltransferase 177_19(ORF43) |
|  |  |  |  |  | DNA methyltransferase 281_19(ORF43) |
|  |  |  |  |  | DNA methyltransferase 544_19(ORF43) |
|  |  |  |  |  | DNA methyltransferase Bk2(ORF37) |
|  |  |  |  |  | DNA methyltransferase EF4(CDS54) |
|  |  |  |  |  | DNA methyltransferase F1(ORF38) |
|  |  |  |  |  | DNA methyltransferase Fi(ORF38) |
|  |  |  |  |  | DNA methyl transferase Fz(ORF43) |
|  |  |  |  |  | DNA methyltransferase Iz(ORF41) |
|  |  |  |  |  | DNA methyl transferase Tb(ORF43) |
|  |  |  |  |  | DNA methyltransferase V_19(ORF43) |
| 41 |  |  |  | DNA methyltransferase | DNA methyltransferase 02_19(ORF43) |
|  |  |  |  |  | DNA methyltransferase 1066_19(ORF43) |
|  |  |  |  |  | DNA methyltransferase 110_19(ORF43) |
|  |  |  |  |  | DNA methyltransferase 11sa_19(ORF43) |
|  |  |  |  |  | DNA methyltransferase 141_19(ORF43) |
|  |  |  |  |  | DNA methyltransferase 177_19(ORF43) |
|  |  |  |  |  | DNA methyltransferase 281_19(ORF43) |
|  |  |  |  |  | DNA methyltransferase 544_19(ORF43) |
|  |  |  |  |  | DNA methyl transferase BK(ORF42) |
|  |  |  |  |  | DNA methyltransferase Bk2(ORF37) |
|  |  |  |  |  | DNA methyltransferase F1(ORF38) |
|  |  |  |  |  | DNA methyltransferase Fi(ORF37) |
|  |  |  |  |  | DNA methyl transferase Fz(ORF43) |
|  |  |  |  |  | DNA methyltransferase Iz(ORF42) |
|  |  |  |  |  | DNA methyl transferase Pr(ORF42) |
|  |  |  |  |  | DNA methyl transferase R/C(ORF42) |
|  |  |  |  |  | DNA methyl transferase S708(ORF42) |
|  |  |  |  |  | DNA methyl transferase Tb(ORF43) |
|  |  |  |  |  | DNA methyltransferase V_19(ORF43) |
|  |  |  |  |  | DNA methyl transferase Wb(ORF42) |
| 42 | 28360 | 27602 | _ | hypothetical protein | hypothetical protein 02_19(ORF44) |
|  |  |  |  |  | hypothetical protein 1066_19(ORF44) |
|  |  |  |  |  | hypothetical protein 110_19(ORF44) |
|  |  |  |  |  | hypothetical protein 11sa_19(ORF44) |
|  |  |  |  |  | hypothetical protein 141_19(ORF44) |
|  |  |  |  |  | hypothetical protein 177_19(ORF44) |
|  |  |  |  |  | hypothetical protein 281_19(ORF44) |
|  |  |  |  |  | hypothetical protein 544_19(ORF44) |
|  |  |  |  |  | hypothetical protein Bk(ORF43) |
|  |  |  |  |  | hypothetical protein Bk2(ORF39) |
|  |  |  |  |  | hypothetical protein EF4(CDS55) |
|  |  |  |  |  | hypothetical protein F1(ORF39) |
|  |  |  |  |  | hypothetical protein Fi(ORF39) |
|  |  |  |  |  | hypothetical protein Fz(ORF44) |
|  |  |  |  |  | hypothetical protein Iz(ORF43) |
|  |  |  |  |  | hypothetical protein Pr(ORF43) |
|  |  |  |  |  | hypothetical protein R/C(ORF43) |
|  |  |  |  |  | hypothetical protein S708(ORF43) |
|  |  |  |  |  | hypothetical protein Tb(ORF44) |
|  |  |  |  |  | hypothetical protein V_19(ORF44) |
|  |  |  |  |  | hypothetical protein Wb(ORF43) |
| 43 | 28547 |  |  | hypothetical protein | hypothetical protein 02_19(ORF45) |
|  |  | 28353 | _ |  | hypothetical protein 1066_19(ORF45) |
|  |  |  |  |  | hypothetical protein 110_19(ORF45) |
|  |  |  |  |  | hypothetical protein 11sa_19(ORF45) |
|  |  |  |  |  | hypothetical protein 141_19(ORF45) |
|  |  |  |  |  | hypothetical protein 177_19(ORF45) |
|  |  |  |  |  | hypothetical protein 281_19(ORF45) |
|  |  |  |  |  | hypothetical protein 544_19(ORF45) |
|  |  |  |  |  | hypothetical protein Bk(ORF44) |
|  |  |  |  |  | hypothetical protein Bk2(ORF40) |
|  |  |  |  |  | hypothetical protein EF4(CDS56) |
|  |  |  |  |  | hypothetical protein F1(ORF40) |
|  |  |  |  |  | hypothetical protein Fi(ORF40) |
|  |  |  |  |  | hypothetical protein Fz(ORF45) |
|  |  |  |  |  | hypothetical protein Iz(ORF44) |
|  |  |  |  |  | hypothetical protein Pr(ORF44) |
|  |  |  |  |  | hypothetical protein R/C(ORF44) |
|  |  |  |  |  | hypothetical protein S708(ORF44) |
|  |  |  |  |  | hypothetical protein Tb(ORF45) |
|  |  |  |  |  | hypothetical protein V_19(ORF45) |
|  |  |  |  |  | hypothetical protein Wb(ORF44) |
| 44 | 28836 | 28579 | - | hypothetical protein | hypothetical protein 02_19(ORF46) |
|  |  |  |  |  | hypothetical protein 1066_19(ORF46) |
|  |  |  |  |  | hypothetical protein 110_19(ORF46) |
|  |  |  |  |  | hypothetical protein 11sa_19(ORF46) |
|  |  |  |  |  | hypothetical protein 141_19(ORF46) |
|  |  |  |  |  | hypothetical protein 177_19(ORF46) |
|  |  |  |  |  | hypothetical protein 281_19(ORF46) |
|  |  |  |  |  | hypothetical protein 544_19(ORF46) |
|  |  |  |  |  | hypothetical protein Bk(ORF45) |
|  |  |  |  |  | hypothetical protein Bk2(ORF41) |
|  |  |  |  |  | hypothetical protein EF4(CDS57) |
|  |  |  |  |  | hypothetical protein F1(ORF41) |
|  |  |  |  |  | hypothetical protein Fi(ORF41) |
|  |  |  |  |  | hypothetical protein Fz(ORF46) |
|  |  |  |  |  | hypothetical protein Iz(ORF45) |
|  |  |  |  |  | hypothetical protein Pr(ORF45) |
|  |  |  |  |  | hypothetical protein R/C(ORF45) |
|  |  |  |  |  | hypothetical protein S708(ORF45) |
|  |  |  |  |  | hypothetical protein Tb(ORF46) |
|  |  |  |  |  | hypothetical protein V_19(ORF46) |
|  |  |  |  |  | hypothetical protein Wb(ORF45) |
| 45 | 29057 | 28899 | _ | hypothetical protein | hypothetical protein Iz(ORF47) |
| 46 | 29302 | 29045 | _ | DUF6378 domain-containing protein | hypothetical protein 02_19(ORF48) |
|  |  |  |  |  | hypothetical protein 1066_19(ORF48) |
|  |  |  |  |  | hypothetical protein 110_19(ORF48) |
|  |  |  |  |  | hypothetical protein 11sa_19(ORF48) |
|  |  |  |  |  | hypothetical protein 141_19(ORF48) |
|  |  |  |  |  | hypothetical protein 177_19(ORF48) |
|  |  |  |  |  | hypothetical protein 281_19(ORF48) |
|  |  |  |  |  | hypothetical protein 544_19(ORF48) |
|  |  |  |  |  | hypothetical protein Bk(ORF47) |
|  |  |  |  |  | hypothetical protein Bk2(ORF43) |
|  |  |  |  |  | hypothetical protein EF4(CDS59) |
|  |  |  |  |  | hypothetical protein F1(ORF43) |
|  |  |  |  |  | hypothetical protein Fi(ORF43) |
|  |  |  |  |  | hypothetical protein Fz(ORF48) |
|  |  |  |  |  | hypothetical protein Iz(ORF48) |
|  |  |  |  |  | DUF6378 domain-containing protein Pr(ORF47) |
|  |  |  |  |  | hypothetical protein R/C(ORF47) |
|  |  |  |  |  | hypothetical protein S708(ORF47) |
|  |  |  |  |  | DUF6378 domain-containing protein Tb(ORF48) |
|  |  |  |  |  | hypothetical protein V_19(ORF48) |
|  |  |  |  |  | hypothetical protein Wb(ORF47) |
| 47 | 29714 | 29289 | _ | type III restriction endonuclease | type III restriction endonuclease 02_19(ORF49) |
|  |  |  |  |  | type III restriction endonuclease 1066_19(ORF49) |
|  |  |  |  |  | type III restriction endonuclease 110_19(ORF49) |
|  |  |  |  |  | type III restriction endonuclease 11sa_19(ORF49) |
|  |  |  |  |  | type III restriction endonuclease 141_19(ORF49) |
|  |  |  |  |  | type III restriction endonuclease 177_19(ORF49) |
|  |  |  |  |  | type III restriction endonuclease 281_19(ORF49) |
|  |  |  |  |  | type III restriction endonuclease 544_19(ORF49) |
|  |  |  |  |  | type III restriction endonuclease BK(ORF48) |
|  |  |  |  |  | type III-restriction endonuclease Bk2(ORF44) |
|  |  |  |  |  | type III restriction endonuclease EF4(CDS60) |
|  |  |  |  |  | type III-restriction endonuclease F1(ORF44) |
|  |  |  |  |  | type III-restriction endonuclease Fi(ORF44) |
|  |  |  |  |  | type III restriction endonuclease Fz(ORF49) |
|  |  |  |  |  | hypothetical protein Iz(ORF49) |
|  |  |  |  |  | type III restriction endonuclease Pr(ORF48) |
|  |  |  |  |  | type III restriction endonuclease R/C(ORF48) |
|  |  |  |  |  | type III restriction endonuclease S708(ORF48) |
|  |  |  |  |  | type III restriction endonuclease Tb(ORF49) |
|  |  |  |  |  | type III restriction endonuclease V_19(ORF49) |
|  |  |  |  |  | type III restriction endonuclease Wb(ORF48) |
| 48 | 31516 | 29711 | _ | DEAD/DEAH box helicase | helicase 02_19(ORF50) |
|  |  |  |  |  | helicase 1066_19(ORF50) |
|  |  |  |  |  | helicase 110_19(ORF50) |
|  |  |  |  |  | helicase 11sa_19(ORF50) |
|  |  |  |  |  | helicase 141_19(ORF50) |
|  |  |  |  |  | helicase 177_19(ORF50) |
|  |  |  |  |  | helicase 281_19(ORF50) |
|  |  |  |  |  | helicase 544_19(ORF50) |
|  |  |  |  |  | helicase BK(ORF49) |
|  |  |  |  |  | helicase Bk2(ORF45) |
|  |  |  |  |  | helicase EF4(CDS61、62、63) |
|  |  |  |  |  | helicase F1(ORF45) |
|  |  |  |  |  | helicase Fi(ORF45) |
|  |  |  |  |  | helicase Fz(ORF50) |
|  |  |  |  |  | helicase Iz(ORF50) |
|  |  |  |  |  | DEAD/DEAH box helicase Pr(ORF49) |
|  |  |  |  |  | helicase R/C(ORF49) |
|  |  |  |  |  | helicase S708(ORF49) |
|  |  |  |  |  | DEAD/DEAH box helicase Tb(ORF50) |
|  |  |  |  |  | helicase V_19(ORF50) |
|  |  |  |  |  | helicase Wb(ORF49) |
| 49 | 32025 | 31519 | _ | DNA-binding protein | DNA-binding protein 02_19(ORF51) |
|  |  |  |  |  | DNA-binding protein 1066_19(ORF51) |
|  |  |  |  |  | DNA-binding protein 110_19(ORF51) |
|  |  |  |  |  | DNA-binding protein 11sa_19(ORF51) |
|  |  |  |  |  | DNA-binding protein 141_19(ORF51) |
|  |  |  |  |  | DNA-binding protein 177_19(ORF51) |
|  |  |  |  |  | DNA-binding protein 281_19(ORF51) |
|  |  |  |  |  | DNA-binding protein 544_19(ORF51) |
|  |  |  |  |  | DNA-binding protein Bk2(ORF46) |
|  |  |  |  |  | DNA-binding HNH endonuclease EF4(CDS64) |
|  |  |  |  |  | DNA-binding protein F1(ORF46) |
|  |  |  |  |  | DNA-binding protein Fi(ORF46) |
|  |  |  |  |  | DNA-binding protein Fz(ORF51) |
|  |  |  |  |  | HNH endonuclease Iz(ORF51) |
|  |  |  |  |  | DNA-binding protein Pr(ORF50) |
|  |  |  |  |  | HNH endonuclease Tb(ORF51) |
|  |  |  |  |  | DNA-binding protein V_19(ORF51) |
| 50 | 32752 | 32126 | _ | DNA-PolB associated exonuclease | DNA-PolB associated exonuclease 02_19(ORF52) |
|  |  |  |  |  | DNA-PolB associated exonuclease 1066_19(ORF52) |
|  |  |  |  |  | DNA-PolB associated exonuclease 110_19(ORF52) |
|  |  |  |  |  | DNA-PolB associated exonuclease 11sa_19(ORF52) |
|  |  |  |  |  | DNA-PolB associated exonuclease 141_19(ORF52) |
|  |  |  |  |  | DNA-PolB associated exonuclease 177_19(ORF52) |
|  |  |  |  |  | DNA-PolB associated exonuclease 281_19(ORF52) |
|  |  |  |  |  | DNA-PolB associated exonuclease 544_19(ORF52) |
|  |  |  |  |  | DNA-PolB associated exonuclease BK(ORF51) |
|  |  |  |  |  | exonuclease Bk2(ORF47) |
|  |  |  |  |  | DNA-PolB associated exonuclease EF4(CDS65) |
|  |  |  |  |  | exonuclease F1(ORF47) |
|  |  |  |  |  | exonuclease Fi(ORF47) |
|  |  |  |  |  | DNA-PolB associated exonuclease Fz(ORF52) |
|  |  |  |  |  | exonuclease Iz(ORF52) |
|  |  |  |  |  | 3'-5' exonuclease Pr(ORF51) |
|  |  |  |  |  | DNA-PolB associated exonuclease R/C(ORF50) |
|  |  |  |  |  | DNA-PolB associated exonuclease S708(ORF51) |
|  |  |  |  |  | 3'-5' exonuclease Tb(ORF52) |
|  |  |  |  |  | DNA-PolB associated exonuclease V_19(ORF52) |
|  |  |  |  |  | DNA-PolB associated exonuclease Wb(ORF51) |
| 51 | 33189 | 32749 | - | hypothetical protein | hypothetical protein 02_19(ORF53) |
|  |  |  |  |  | hypothetical protein 1066_19(ORF53) |
|  |  |  |  |  | hypothetical protein 110_19(ORF53) |
|  |  |  |  |  | hypothetical protein 11sa_19(ORF53) |
|  |  |  |  |  | hypothetical protein 141_19(ORF53) |
|  |  |  |  |  | hypothetical protein 177_19(ORF53) |
|  |  |  |  |  | hypothetical protein 281_19(ORF53) |
|  |  |  |  |  | hypothetical protein 544_19(ORF53) |
|  |  |  |  |  | hypothetical protein Bk(ORF52) |
|  |  |  |  |  | hypothetical protein Bk2(ORF48) |
|  |  |  |  |  | hypothetical protein EF4(CDS66) |
|  |  |  |  |  | hypothetical protein F1(ORF48) |
|  |  |  |  |  | hypothetical protein Fi(ORF48) |
|  |  |  |  |  | hypothetical protein Fz(ORF53) |
|  |  |  |  |  | hypothetical protein Iz(ORF53) |
|  |  |  |  |  | hypothetical protein Pr(ORF52) |
|  |  |  |  |  | hypothetical protein R/C(ORF52) |
|  |  |  |  |  | hypothetical protein S708(ORF52) |
|  |  |  |  |  | hypothetical protein Tb(ORF53) |
|  |  |  |  |  | hypothetical protein V_19(ORF53) |
|  |  |  |  |  | hypothetical protein Wb(ORF52) |
| 52 | 33844 | 33239 | - | single strand DNA binding protein | hypothetical protein 02_19(ORF54) |
|  |  |  |  |  | hypothetical protein 1066_19(ORF54) |
|  |  |  |  |  | hypothetical protein 110_19(ORF54) |
|  |  |  |  |  | hypothetical protein 11sa_19(ORF54) |
|  |  |  |  |  | hypothetical protein 141_19(ORF54) |
|  |  |  |  |  | hypothetical protein 177_19(ORF54) |
|  |  |  |  |  | hypothetical protein 281_19(ORF54) |
|  |  |  |  |  | hypothetical protein 544_19(ORF54) |
|  |  |  |  |  | hypothetical protein Bk(ORF53) |
|  |  |  |  |  | hypothetical protein Bk2(ORF49) |
|  |  |  |  |  | hypothetical protein EF4(CDS67) |
|  |  |  |  |  | hypothetical protein F1(ORF49) |
|  |  |  |  |  | hypothetical protein Fi(ORF49) |
|  |  |  |  |  | hypothetical protein Fz(ORF54) |
|  |  |  |  |  | hypothetical protein Iz(ORF54) |
|  |  |  |  |  | single strand DNA binding protein Pr(ORF53) |
|  |  |  |  |  | hypothetical protein R/C(ORF53) |
|  |  |  |  |  | hypothetical protein S708(ORF53) |
|  |  |  |  |  | single strand DNA binding protein Tb(ORF54) |
|  |  |  |  |  | hypothetical protein V_19(ORF54) |
|  |  |  |  |  | hypothetical protein Wb(ORF53) |
| 53 | 34131 | 33847 | - | hypothetical protein | hypothetical protein 02_19(ORF55) |
|  |  |  |  |  | hypothetical protein 1066_19(ORF55) |
|  |  |  |  |  | hypothetical protein 110_19(ORF55) |
|  |  |  |  |  | hypothetical protein 11sa_19(ORF55) |
|  |  |  |  |  | hypothetical protein 141_19(ORF55) |
|  |  |  |  |  | hypothetical protein 177_19(ORF55) |
|  |  |  |  |  | hypothetical protein 281_19(ORF55) |
|  |  |  |  |  | hypothetical protein 544_19(ORF55) |
|  |  |  |  |  | hypothetical protein Bk(ORF54) |
|  |  |  |  |  | hypothetical protein Bk2(ORF50) |
|  |  |  |  |  | hypothetical protein EF4(CDS68) |
|  |  |  |  |  | hypothetical protein F1(ORF50) |
|  |  |  |  |  | hypothetical protein Fi(ORF50) |
|  |  |  |  |  | hypothetical protein Fz(ORF55) |
|  |  |  |  |  | hypothetical protein Iz(ORF55) |
|  |  |  |  |  | hypothetical protein Pr(ORF54) |
|  |  |  |  |  | hypothetical protein R/C(ORF54) |
|  |  |  |  |  | hypothetical protein S708(ORF54) |
|  |  |  |  |  | hypothetical protein Tb(ORF55) |
|  |  |  |  |  | hypothetical protein V_19(ORF55) |
|  |  |  |  |  | hypothetical protein Wb(ORF54) |
| 54 | 34877 | 34131 | - | Sak4-like ssDNA annealing protein | hypothetical protein 02_19(ORF56) |
|  |  |  |  |  | hypothetical protein 1066_19(ORF56) |
|  |  |  |  |  | hypothetical protein 110_19(ORF56) |
|  |  |  |  |  | hypothetical protein 11sa_19(ORF56) |
|  |  |  |  |  | hypothetical protein 141_19(ORF56) |
|  |  |  |  |  | hypothetical protein 177_19(ORF56) |
|  |  |  |  |  | hypothetical protein 281_19(ORF56) |
|  |  |  |  |  | hypothetical protein 544_19(ORF56) |
|  |  |  |  |  | hypothetical protein Bk(ORF55) |
|  |  |  |  |  | hypothetical protein Bk2(ORF51) |
|  |  |  |  |  | AAA family ATPase EF4(CDS69) |
|  |  |  |  |  | hypothetical protein F1(ORF51) |
|  |  |  |  |  | hypothetical protein Fi(ORF51) |
|  |  |  |  |  | hypothetical protein Fz(ORF56) |
|  |  |  |  |  | helicase subunit Iz(ORF56) |
|  |  |  |  |  | Sak4-like ssDNA annealing protein Pr(ORF55) |
|  |  |  |  |  | hypothetical protein R/C(ORF55) |
|  |  |  |  |  | hypothetical protein S708(ORF55) |
|  |  |  |  |  | Sak4-like ssDNA annealing protein Tb(ORF56) |
|  |  |  |  |  | hypothetical protein V_19(ORF56) |
|  |  |  |  |  | hypothetical protein Wb(ORF55) |
| 55 | 35032 | 35160 | + | hypothetical protein | hypothetical protein 02_19(ORF57) |
|  |  |  |  |  | hypothetical protein 1066_19(ORF57) |
|  |  |  |  |  | hypothetical protein 110_19(ORF57) |
|  |  |  |  |  | hypothetical protein 11sa_19(ORF57) |
|  |  |  |  |  | hypothetical protein 141_19(ORF57) |
|  |  |  |  |  | hypothetical protein 177_19(ORF57) |
|  |  |  |  |  | hypothetical protein 281_19(ORF57) |
|  |  |  |  |  | hypothetical protein 544_19(ORF57) |
|  |  |  |  |  | hypothetical protein Bk(ORF56) |
|  |  |  |  |  | hypothetical protein Bk2(ORF52) |
|  |  |  |  |  | hypothetical protein EF4(CDS70) |
|  |  |  |  |  | hypothetical protein F1(ORF52) |
|  |  |  |  |  | hypothetical protein Fi(ORF52) |
|  |  |  |  |  | hypothetical protein Fz(ORF57) |
|  |  |  |  |  | hypothetical protein Pr(ORF56) |
|  |  |  |  |  | hypothetical protein R/C(ORF56) |
|  |  |  |  |  | hypothetical protein S708(ORF56) |
|  |  |  |  |  | hypothetical protein Tb(ORF57) |
|  |  |  |  |  | hypothetical protein V_19(ORF57) |
|  |  |  |  |  | hypothetical protein Wb(ORF56) |
| 56 | 37654 | 35312 | - | bifunctional DNA primase/polymerase | bifunctional DNA primase/polymerase 02_19(ORF58) |
|  |  |  |  |  | bifunctional DNA primase/polymerase 1066_19(ORF58) |
|  |  |  |  |  | bifunctional DNA primase/polymerase 110_19(ORF58) |
|  |  |  |  |  | bifunctional DNA primase/polymerase 11sa_19(ORF58) |
|  |  |  |  |  | bifunctional DNA primase/polymerase 141_19(ORF58) |
|  |  |  |  |  | bifunctional DNA primase/polymerase 177_19(ORF58) |
|  |  |  |  |  | bifunctional DNA primase/polymerase 281_19(ORF58) |
|  |  |  |  |  | bifunctional DNA primase/polymerase 544_19(ORF58) |
|  |  |  |  |  | bifunctional DNA primase/polymerase BK(ORF57) |
|  |  |  |  |  | DNA primase/polymerase Bk2(ORF53) |
|  |  |  |  |  | bifunctional DNA primase/polymerase EF4(CDS71、72) |
|  |  |  |  |  | DNA primase/helicase F1(ORF53) |
|  |  |  |  |  | DNA primase/polymerase Fi(ORF53) |
|  |  |  |  |  | bifunctional DNA primase/polymerase Fz(ORF58) |
|  |  |  |  |  | DNA primase Iz(ORF57、58) |
|  |  |  |  |  | DNA primase Pr(ORF57) |
|  |  |  |  |  | bifunctional DNA primase/polymerase R/C(ORF57) |
|  |  |  |  |  | bifunctional DNA primase/polymerase S708(ORF57) |
|  |  |  |  |  | DNA primase Tb(ORF58) |
|  |  |  |  |  | bifunctional DNA primase/polymerase V_19(ORF58) |
|  |  |  |  |  | bifunctional DNA primase/polymerase Wb(ORF57) |
